# Validation-Constrained Adaptive Residual Fusion Framework for Low-Latitude TEC Prediction in GNSS Ionospheric Sensing

**DOI:** 10.3390/s26165104

**Published:** 2026-08-12

**Authors:** Mengjia Bai, Yuanfa Ji, Xiyan Sun, Wenbin Liang, Kamarul Hawari Bin Ghazali

**Affiliations:** 1 School of Information and Communication, Guilin University of Electronic Technology, Guilin 541004, China; baimengjia@mails.guet.edu.cn (M.B.); liangwenbin@guat.edu.cn (W.L.); 2 Guangxi Key Laboratory of Precision Navigation Technology and Application, Guilin University of Electronic Technology, Guilin 541004, China; 3 China–ASEAN International Joint Laboratory for Spatial Information and Intelligent Location Services, Guilin 541004, China; 4 GUET-Nanning E-Tech Research Institute Co., Ltd., Nanning 530031, China; 5 Faculty of Electrical and Electronics Engineering Technology, Universiti Malaysia Pahang Al-Sultan Abdullah, Pekan Campus, Pekan 26600, Pahang, Malaysia; kamarul@umpsa.edu.my

**Keywords:** low-latitude ionosphere, GNSS ionospheric sensing, TEC prediction, residual correction, adaptive residual fusion, grouped TreeSHAP, XGBoost, CNN-BiLSTM

## Abstract

Accurate prediction of low-latitude total electron content, or TEC, is important for GNSS ionospheric sensing because pronounced diurnal variation and sensitivity to solar and geomagnetic activity make TEC highly nonlinear and nonstationary. This study proposes the RC-XGB-CNN-BiLSTM validation-constrained adaptive residual-fusion framework for one-hour-ahead TEC prediction. XGBoost provides the primary estimate from historical TEC, local-time factors, and solar–terrestrial inputs, while CNN-BiLSTM learns residuals generated from chronologically ordered out-of-fold predictions. Validation performance determines the residual-use mode and compensation strength. Experiments cover multivariate inputs, contrasting solar and geomagnetic activity conditions, module-wise ablation, and additional low-latitude grid points. In the 2019 multivariate experiment at 20° N, 110° E, the framework achieves an RMSE of 0.67 TECU, 20.2% lower than XGBoost, with concurrent reductions in MAE and P90AE. It also achieves the lowest errors among the compared models in both solar-activity experiments and at all three additional grid points. Grouped TreeSHAP identifies recent TEC as the dominant predictive information, while residual-gain analysis shows that validation-controlled fusion adapts correction to activity conditions and retains the primary prediction when additional correction is not beneficial. These results support validation-constrained residual fusion as an effective approach for low-latitude TEC prediction.

## 1. Introduction

Global Navigation Satellite Systems (GNSS) support positioning, navigation, timing, geodetic observation, and ionospheric monitoring, yet their signals are strongly affected by the dispersive ionosphere. Total electron content (TEC) provides a compact measure of the integrated electron density along the signal path and is widely used to characterize ionospheric effects on GNSS observations [1,2]. Short-term TEC prediction is particularly challenging at low latitudes, where equatorial electrodynamics, the equatorial ionization anomaly, and pronounced longitudinal and local-time dependence generate strong spatial gradients and day-to-day variability [3]. Solar-radiation variability and geomagnetic disturbances further modify TEC through coupled solar–wind–magnetosphere–ionosphere processes [3,4]. Accurate short-term prediction in this region is therefore important for GNSS ionospheric sensing and for characterizing rapidly varying low-latitude ionospheric conditions.

Data-driven TEC forecasting has evolved from early ionospheric mapping and neural-network approaches toward models that combine recent ionospheric-state information with external drivers. Schaer established an important GNSS-based foundation for global ionospheric mapping and prediction, while early neural-network studies demonstrated the feasibility of short-horizon and map-based TEC forecasting [5,6,7,8]. Subsequent studies incorporated recent TEC, local time, solar activity, geomagnetic indices, and interplanetary quantities as complementary predictors. Zewdie et al. used variable-importance analysis to select solar–terrestrial parameters for low-latitude TEC forecasting; Zhou et al. incorporated solar and geomagnetic indices, temporal descriptors, and historical TEC information into ensemble-learning models for one-hour-ahead TEC prediction; and Liu et al. combined historical ionospheric information with solar, geomagnetic, and temporal inputs for short-horizon global TEC prediction [9,10,11]. These studies support the combined use of historical TEC and physically relevant auxiliary information and motivate a structured assessment of their relative predictive contributions.

Machine-learning and deep-learning methods have substantially expanded the ability to represent nonlinear and time-dependent TEC variability. Empirical ionospheric models remain useful climatological references, but their representation of rapid departures from the background state is limited [12]. A recent review identifies geomagnetic disturbances, input representation, and limited physical interpretability as continuing challenges for data-driven ionospheric modeling [13]. Tree-based and recurrent models have demonstrated short-horizon regional and global forecasting using ionospheric and solar–terrestrial information, while LSTM and ConvLSTM architectures have been extended to regional and global spatiotemporal TEC prediction [9,11,14,15]. Storm-oriented studies further show that multichannel ConvLSTM and ensemble learning can improve the representation of disturbed-period TEC variability [16,17].

Recent studies have further extended TEC forecasting toward Transformer, graph-based, and hybrid architectures. Autocorrelation-based Transformers, graph-enabled spatiotemporal models, and TECFormer have been developed to represent long-range temporal dependence, spatial relationships, and periodic TEC evolution [18,19,20]. Hybrid approaches have combined graph or convolutional feature extraction with recurrent temporal modeling for regional and storm-time forecasting, while ED-Autoformer incorporates TEC together with solar-wind, IMF, and geomagnetic information for global prediction under different activity levels [21,22,23]. These developments broaden the representation capacity of TEC forecasting models; however, increasing architectural complexity or adding another prediction stage does not by itself ensure a transferable improvement.

Despite these advances, three issues remain important for residual-based short-term TEC forecasting. First, residual targets derived from in-sample primary predictions can contain fitting-specific error patterns and may therefore provide an optimistic representation of the residual structure available in later periods. Second, a learned residual is not necessarily beneficial for every sample; direct or fixed-strength correction can increase the prediction error when the estimated residual has an unreliable sign or magnitude. Third, feature attribution of the primary predictor alone does not reveal whether the residual-correction stage is beneficial or under which ionospheric conditions it contributes to error reduction. Explainable machine-learning methods provide useful tools for examining predictive information pathways [24,25,26], but residual-based forecasting additionally requires explicit evaluation of the correction outcome. These considerations motivate a framework that combines temporally transferable residual construction, validation-controlled residual use, and complementary diagnostics for the primary and residual stages.

To address these issues, this study develops a validation-constrained adaptive residual-fusion framework for one-hour-ahead low-latitude TEC prediction. XGBoost provides the primary estimate from historical TEC, local-time information, and solar–terrestrial inputs, while a CNN-BiLSTM branch learns residual targets generated from chronologically ordered out-of-fold predictions; validation performance then determines whether and how strongly the residual estimate is incorporated. The contributions are threefold. First, chronological out-of-fold prediction is used to construct residual-learning targets while preserving the temporal order of the forecasting problem. Second, a validation-constrained adaptive fusion strategy selects the residual-use mode and compensation strength, allowing the primary prediction to be retained when additional correction is not beneficial. Third, grouped TreeSHAP attribution is combined with residual-correction gain analysis to characterize both the information used by the primary predictor and the conditions under which residual compensation improves prediction. The framework is evaluated through multivariate-input experiments, contrasting solar and geomagnetic activity conditions, module-wise ablation, and spatial validation at multiple low-latitude grid points.

## 2. Data and Methods

### 2.1. Data Selection

TEC data at the 20° N, 110° E grid point were extracted from the final CODE Global Ionosphere Map (GIM) product [5,27]. The CODE GIM has a spatial grid spacing of 2.5° in latitude and 5° in longitude, and the TEC series used in this study has a temporal resolution of 1 h. The selected grid point lies in a low-latitude ionospheric region where TEC is strongly affected by solar radiation, geomagnetic disturbances, and low-latitude ionospheric dynamics. The TEC series at this location exhibits clear diurnal periodicity, nonlinearity, and nonstationarity, providing a representative setting for evaluating prediction performance under variable ionospheric conditions.

To characterize external forcing associated with TEC variability, the model includes the F10.7 solar radio flux, the Dst and ap geomagnetic indices, and the interplanetary magnetic field (IMF) components Bx, By, and Bz as exogenous inputs. F10.7 is used as a proxy for the solar-radiation background; Dst and ap characterize ring-current variation and planetary geomagnetic activity, respectively. The IMF components describe the magnetic-field configuration relevant to solar-wind–magnetosphere–ionosphere coupling. The F10.7 solar radio flux, geomagnetic indices, and IMF components were obtained from NASA/GSFC SPDF OMNIWeb [28,29] and temporally aligned with the TEC series.

TEC exhibits pronounced diurnal variability, and its phase varies with longitude. For each grid point, universal time (UT) is converted to local time (LT) according to longitude λ:

(1) $$LT=\mathrm{mod}\left(UT+\frac{\lambda }{15},24\right).$$

The local-time harmonic factors are then defined as

(2) $$\left\{\begin{array}{cc} HDs & =\sin\left(\frac{2\pi LT}{24}\right),\\ HDc & =\cos\left(\frac{2\pi LT}{24}\right). \end{array}\right.$$

Here, λ denotes longitude in degrees east and LT denotes local time in hours. HDs and HDc are the sine and cosine components of the local-time phase, respectively.

Historical TEC, local-time harmonics, F10.7, geomagnetic indices, and IMF components constitute the model input variables summarized in Table 1.

### 2.2. Data Preprocessing

Because TEC observations and the external input quantities do not have exactly the same original temporal resolution, the 1 h TEC sampling interval is used as the unified time base. Hourly IMF components and Dst are matched to the TEC series by time stamp. For temporal alignment, each 3 h ap observation is assigned to the three corresponding hourly samples, and each daily F10.7 observation is assigned to all hourly samples within the same day. This treatment preserves the original temporal meaning of the indices while providing inputs on the common 1 h TEC time grid.

During quality control, abnormal fill values in the external input records and TEC sequence are first set to missing values. TEC observations smaller than 0 are also regarded as physically unreasonable abnormal values and set to missing. Short gaps are filled by linear interpolation with a maximum consecutive interpolation interval of 2 h. Let $x_{a}$ and $x_{b}$ be valid observations at the two ends of a missing interval, and let $t_{a}<t<t_{b}$ be the interpolation time. The interpolated value is

(3) $${\hat{x}}_{t}=x_{a}+\frac{t-t_{a}}{t_{b}-t_{a}}\left(x_{b}-x_{a}\right),\;t_{b}-t_{a}\leq 2\;h.$$

This treatment only locally repairs short gaps and avoids imposing a strong linear-continuity assumption on long missing intervals, thereby reducing potential disturbance to the original dynamic structure of the sequence.

To suppress short-duration noise while preserving forecasting causality, wavelet-threshold denoising is performed independently within each 24 h historical window. Each reconstructed input is therefore generated exclusively from observations available through the input endpoint, while the multiscale decomposition retains the slowly varying TEC background and short-term fluctuation structure required for one-hour-ahead prediction. For the one-hour-ahead target at t+1, the denoised historical input is constructed from the TEC observations available through time *t*:

(4) $$Y_{t}=\left\{{\mathrm{TEC}}_{t-L+1},{\mathrm{TEC}}_{t-L+2},\ldots ,{\mathrm{TEC}}_{t}\right\}.$$

Here, L=24 h, so the historical window spans t−23 to *t* and preserves forecast causality.

The db8 basis, SURE threshold and soft shrinkage are used to obtain a smooth reconstruction while reducing local high-frequency noise. Wavelet multiresolution decomposition separates low-frequency background variations and high-frequency disturbance information at different scales [30], and wavelet-denoising strategies have also been used in TEC prediction models to reduce the effect of random noise on subsequent learning [31]. Let the original TEC sequence be $y_{t}$. After *J*-level wavelet decomposition, it is written as

(5) $$y_{t}=A_{J}\left(t\right)+\sum_{j=1}^{J}D_{j}\left(t\right),$$

where $A_{J}\left(t\right)$ is the low-frequency approximation component and $D_{j}\left(t\right)$ is the detail component at level *j*. For any detail coefficient $d_{j,k}$, soft-thresholding is defined as

(6) $${\tilde{d}}_{j,k}=\mathrm{sgn}\left(d_{j,k}\right)\max\left(\left|d_{j,k}\right|-\lambda ,0\right),$$

where λ is the adaptive threshold. The denoised TEC sequence ${\tilde{y}}_{t}$ is reconstructed from the low-frequency approximation coefficients and thresholded detail coefficients. The denoised TEC is used only as an input feature, whereas prediction labels and evaluation metrics use the original TEC:

(7) $$x_{t,l}^{TEC}={\tilde{\mathrm{TEC}}}_{t-l},\;l=0,1,\ldots ,L-1,\;y_{t+1}={\mathrm{TEC}}_{t+1}.$$

Here, $x_{t,l}^{TEC}$ denotes the denoised TEC input at lag *l*, while the prediction label is the original TEC observation at t+1.

### 2.3. Supervised Sample Construction and Feature Representation

A sliding time window is used to convert the continuous TEC sequence into supervised-learning samples. Let *t* denote the endpoint of the available input information, let L=24 h be the historical window length, and let Δ=1 h be the prediction horizon. The supervised mapping is

(8) $${\hat{y}}_{t+1}=f\left(x_{t}\right),\;y_{t+1}={\mathrm{TEC}}_{t+1}.$$

Here, $x_{t}$ contains information available at or before *t*, and $y_{t+1}$ is the original TEC observation one hour later.

For multivariate input, the exogenous input vector is defined as

(9) $$e_{t}={\left[HDs_{t},HDc_{t},Bx_{t},By_{t},Bz_{t},Dst_{t},ap_{t},{F10.7}_{t}\right]}^{T}.$$

During sample construction, the exogenous context spans t−2 to *t*, and the TEC branch uses the denoised observations from t−23 to *t*. The multivariate and univariate input vectors are therefore

(10) $$x_{t}^{multi}={\left[e_{t-2}^{T},e_{t-1}^{T},e_{t}^{T},{\tilde{\mathrm{TEC}}}_{t-23},{\tilde{\mathrm{TEC}}}_{t-22},\ldots ,{\tilde{\mathrm{TEC}}}_{t}\right]}^{T}.$$

(11) $$x_{t}^{uni}={\left[{\tilde{\mathrm{TEC}}}_{t-23},{\tilde{\mathrm{TEC}}}_{t-22},\ldots ,{\tilde{\mathrm{TEC}}}_{t}\right]}^{T}.$$

All valid samples are ordered chronologically and divided into training, validation and test sets at approximate proportions of 80%, 10% and 10%, without random shuffling. The corresponding sample counts are reported with each experimental dataset in Section 3.

To remove the influence of different physical units on model training, only training-set statistics are used to standardize inputs and outputs. Let $\mu_{x}$ and $\sigma_{x}$ be the mean and standard deviation of training-set input features, and let $\mu_{y}$ and $\sigma_{y}$ be the mean and standard deviation of training-set targets. Standardization and inverse standardization are performed as

(12) $$x_{t}^{\prime }=\frac{x_{t}-\mu_{x}}{\sigma_{x}},\;y_{t+1}^{\prime }=\frac{y_{t+1}-\mu_{y}}{\sigma_{y}}.$$

(13) $${\hat{y}}_{t+1}={\hat{y}}_{t+1}^{\prime }\sigma_{y}+\mu_{y}.$$

This procedure ensures that validation and test sets do not participate in normalization-parameter estimation, avoiding data leakage caused by full-sample statistics.

### 2.4. Residual-Correction Fusion Prediction Framework for GNSS TEC Sensing Data

**Figure 1:**
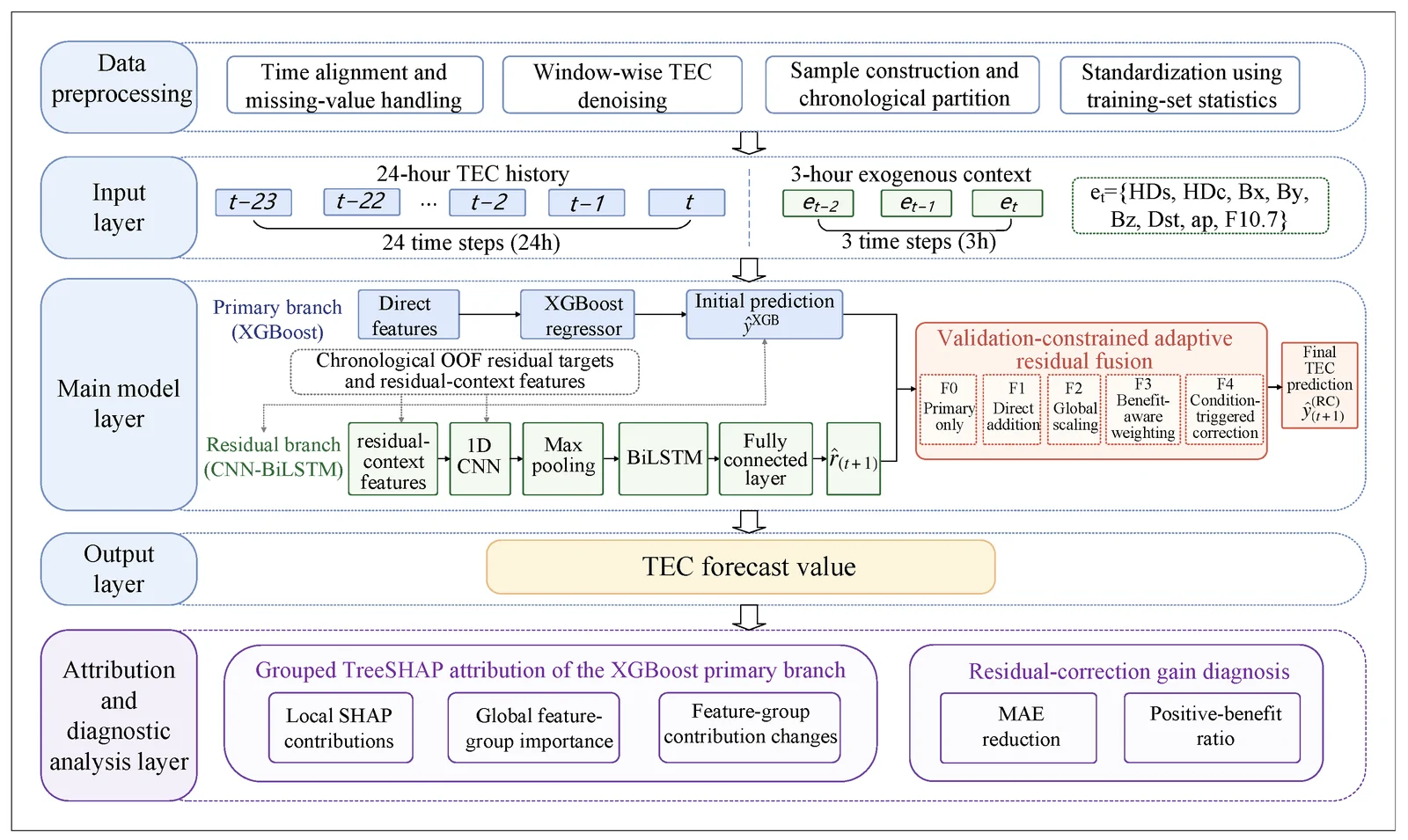
Overall workflow of the validation-constrained adaptive residual-fusion framework for low-latitude TEC prediction.

The proposed framework consists of four successive stages: XGBoost primary prediction, chronological out-of-fold residual generation, CNN-BiLSTM residual learning, and validation-constrained adaptive residual fusion. Grouped TreeSHAP attribution and residual-correction gain analysis characterize the information basis of the primary prediction and the effectiveness of the correction stage (Figure 1).

#### 2.4.1. BiLSTM Baseline Model

BiLSTM is used as a baseline to model temporal dependence in the historical TEC sequence. By processing the sequence in both temporal directions within the input window, it provides a bidirectional representation of periodic variation and short-term fluctuations.

Let the standardized input sequence of the *t*th prediction sample be

(14) $$X_{t}^{\prime }=\left[x_{t-L+1}^{\prime },x_{t-L+2}^{\prime },\ldots ,x_{t}^{\prime }\right],$$

where *L* is the historical window length and $x_{\tau }^{\prime }$ is the standardized input feature vector at time τ. The hidden states extracted by the forward and backward LSTM are

(15) $$\begin{array}{cc} {\vec{h}}_{\tau } & =LSTM_{f}\left(x_{\tau }^{\prime },{\vec{h}}_{\tau -1}\right),\\ {\overset{\leftarrow }{h}}_{\tau } & =LSTM_{b}\left(x_{\tau }^{\prime },{\overset{\leftarrow }{h}}_{\tau +1}\right). \end{array}$$

Here, $LSTM_{f}\left(\cdot \right)$ and $LSTM_{b}\left(\cdot \right)$ denote the forward and backward LSTM mappings, respectively, and the two hidden states correspond to the two directions. The bidirectional temporal representation is obtained by concatenating the two hidden states:

(16) $$h_{\tau }^{bi}=\left[{\vec{h}}_{\tau };{\overset{\leftarrow }{h}}_{\tau }\right].$$

In this implementation, the terminal bidirectional hidden state is used as the comprehensive temporal representation of the prediction sample. A Dropout layer and a fully connected regression layer then output the TEC prediction:

(17) $${\hat{y}}_{t+1}^{\prime }=W_{2}\phi \left(W_{1}\mathrm{Dropout}\left(h_{t}^{bi}\right)+b_{1}\right)+b_{2},$$

where ϕ(·) is the ReLU activation function, $W_{1}$, $W_{2}$, $b_{1}$ and $b_{2}$ are fully connected layer parameters, and ${\hat{y}}_{t+1}^{\prime }$ is the predicted value in standardized space.

#### 2.4.2. CNN-BiLSTM Baseline Model

CNN-BiLSTM introduces a one-dimensional convolutional layer before BiLSTM to extract local neighborhood patterns in the input feature sequence [32]. Let $X\in R^{l\times d}$ be the input sequence. The output of the *m*th channel of the one-dimensional convolutional layer is

(18) $$c_{m}=\phi \left(\mathrm{BN}\left(w_{m}*X+b_{m}\right)\right),$$

where ∗ denotes one-dimensional convolution, BN(·) denotes batch normalization, and ϕ(·) denotes the ReLU activation function. The convolutional output is passed through max pooling and Dropout and then fed into the bidirectional LSTM:

(19) $$P=\mathrm{MaxPool}\left({\left\{c_{m}\right\}}_{m=1}^{M}\right),\;h_{t}^{cnn-bi}=\mathrm{BiLSTM}\left(P\right).$$

The standardized CNN-BiLSTM output is

(20) $${\hat{y}}_{t+1}^{\prime ,\text{CNN-BiLSTM}}=W_{2}\phi \left(W_{1}h_{t}^{\text{cnn-bi}}+b_{1}\right)+b_{2}.$$

The output is transformed back to the original TEC scale using Equation (13) before performance evaluation. The convolutional stage is designed to represent local abrupt changes, peak–trough transitions, and neighboring feature interactions before recurrent temporal modeling, while the BiLSTM captures temporal dependencies within the sequence.

#### 2.4.3. XGBoost Primary Prediction Module

To exploit the structured information in historical TEC state, local-time factors, and solar–terrestrial inputs, XGBoost is used as the primary predictor [33]. For the input endpoint *t*, the XGBoost primary prediction is

(21) $${\hat{y}}_{t+1}^{XGB}=f_{XGB}\left(x_{t}\right),$$

where $f_{XGB}\left(\cdot \right)$ denotes the gradient-boosted model consisting of multiple regression trees. Its additive-tree prediction is

(22) $${\hat{y}}_{t+1}^{XGB}=\sum_{m=1}^{M}f_{m}\left(x_{t}\right),\;f_{m}\in F.$$

where *M* is the number of regression trees, $f_{m}$ is the *m*th regression tree and F is the regression-tree function space. The optimization objective in the *m*th iteration is

(23) $$L^{\left(m\right)}=\sum_{i=1}^{N}l\left(y_{i},{\hat{y}}_{i}^{\left(m-1\right)}+f_{m}\left(x_{i}\right)\right)+\Omega \left(f_{m}\right),$$

where l(·) is the squared-error loss function and $\Omega (f_{m})$ is the model-complexity regularization term:

(24) $$\Omega \left(f_{m}\right)=\gamma K+\frac{1}{2}\lambda \sum_{k=1}^{K}w_{k}^{2}.$$

Here, *K* is the number of leaf nodes, $w_{k}$ is the weight of the *k*th leaf node, and γ and λ are regularization parameters. Through this objective, XGBoost learns the nonlinear mapping between input features and TEC while controlling model complexity.

#### 2.4.4. CNN-BiLSTM Residual-Correction Module

Although XGBoost can characterize the nonlinear relationship between TEC and physically meaningful structured features, its output may still contain local temporal bias. To compensate for dynamic errors not captured by the primary predictor, a CNN-BiLSTM residual-correction module models the XGBoost primary-prediction residual:

(25) $$r_{t+1}=y_{t+1}-{\hat{y}}_{t+1}^{XGB}.$$

The residual targets used to train CNN-BiLSTM are generated through chronological out-of-fold prediction within the training period. In each block, XGBoost is fitted using only the preceding samples and then predicts the immediate following block. The resulting residuals are concatenated in temporal order to form the residual-learning set. Consequently, every residual target is obtained from an observation that was not used to fit the corresponding XGBoost model, which improves the temporal transferability of the learned correction.

The residual branch uses predefined contextual descriptors that characterize the primary-prediction level, recent TEC state, short-term differences, and within-window variability. These descriptors complement the original predictor input by emphasizing amplitude and fluctuation patterns associated with primary-prediction errors. The residual-branch input is summarized as

(26) $$z_{t}={\left[x_{t},{\hat{y}}_{t+1}^{XGB},\left|{\hat{y}}_{t+1}^{XGB}\right|,{\left({\hat{y}}_{t+1}^{XGB}\right)}^{2},s_{t}^{TEC},s_{t}^{fluc}\right]}^{T}.$$

The residual-branch input consists of the original predictor vector and 12 predefined contextual descriptors. These descriptors include three primary-prediction descriptors, namely the XGBoost prediction, its absolute value and its squared value; five historical TEC state descriptors, including the mean, standard deviation, range, latest first difference and latest second difference within the 24 h historical window; and four fluctuation descriptors, including the latest value, mean, standard deviation and range of the raw-minus-denoised TEC component. Consequently, the residual-branch input contains 36 dimensions under the univariate setting and 60 dimensions under the multivariate setting.

Here, $s_{t}^{TEC}$ denotes historical TEC state statistics and $s_{t}^{fluc}$ denotes TEC fluctuation statistics. The learning objective of residual CNN-BiLSTM is

(27) $${\hat{r}}_{t+1}=g_{CNN-BiLSTM}\left(z_{t}\right),\;\min_{g}\sum_{t\in D_{train}}{\left(r_{t+1}-{\hat{r}}_{t+1}\right)}^{2}.$$

If direct residual correction is adopted, the final predicted value is

(28) $${\hat{y}}_{t+1}^{direct}={\hat{y}}_{t+1}^{XGB}+{\hat{r}}_{t+1}.$$

In this structure, the CNN layer captures short-term local fluctuations, and the BiLSTM layer represents forward and backward dependence within the historical window. Together, they provide temporal compensation for XGBoost primary-prediction errors.

#### 2.4.5. Validation-Constrained Adaptive Residual-Fusion Strategy

Residual predictions are incorporated through five candidate outputs: the XGBoost primary prediction (F0), direct residual addition (F1), globally scaled residual addition (F2), benefit-aware residual weighting (F3) and condition-triggered residual correction (F4). The candidates share the same primary prediction and residual estimate and differ only in how the residual is used.

Let ${\hat{y}}_{t+1}^{XGB}$ and ${\hat{r}}_{t+1}$ denote the primary prediction and residual estimate, respectively. The first three candidates are defined as

(29) $${\hat{y}}_{t+1}^{\left(0\right)}={\hat{y}}_{t+1}^{XGB},\;{\hat{y}}_{t+1}^{\left(1\right)}={\hat{y}}_{t+1}^{XGB}+{\hat{r}}_{t+1},\;{\hat{y}}_{t+1}^{\left(2\right)}={\hat{y}}_{t+1}^{XGB}+\alpha_{g}{\hat{r}}_{t+1},$$

where $\alpha_{g}$ is a scenario-specific global scaling coefficient selected using the validation set.

For F3, the benefit label is defined from the absolute-error improvement of direct correction:

(30) $$b_{t}=I\left[e_{t+1}^{\left(0\right)}-e_{t+1}^{\left(1\right)}>\max\left(\delta_{0},\delta_{1}e_{t+1}^{\left(0\right)}\right)\right],$$

where $e_{t+1}^{\left(0\right)}$ and $e_{t+1}^{\left(1\right)}$ are the absolute errors of F0 and F1. A benefit classifier trained on the out-of-fold training samples estimates $p_{t}=P\left(b_{t}=1\right)$. The benefit-aware candidate is

(31) $${\hat{y}}_{t+1}^{\left(3\right)}={\hat{y}}_{t+1}^{XGB}+p_{t}{\hat{r}}_{t+1}.$$

For F4, a risk score $q_{t}$ is calculated from the predicted residual magnitude, recent TEC differences and historical fluctuation statistics. The trigger is

(32) $$m_{t}=I\left[q_{t}\geq Q_{\rho }\left(q_{train}\right)\right],$$

where $Q_{\rho }\left(q_{train}\right)$ is the ρ-quantile estimated from the training data. The condition-triggered candidate is

(33) $${\hat{y}}_{t+1}^{\left(4\right)}={\hat{y}}_{t+1}^{XGB}+m_{t}\;\mathrm{clip}\left(\alpha_{c}{\hat{r}}_{t+1},-c\sigma_{r},c\sigma_{r}\right),$$

where $\alpha_{c}$ is the residual scale, *c* is the clipping coefficient and $\sigma_{r}$ is the standard deviation of the training residual estimates.

For each experimental scenario, all five candidates are evaluated on the same validation set. Let $R_{val}^{\left(k\right)}$ denote the validation RMSE of F*k*. The selected candidate and final prediction are

(34) $$k^{*}={\mathrm{argmin}}_{k\in \{0,1,2,3,4\}}R_{val}^{\left(k\right)},\;{\hat{y}}_{t+1}^{RC}={\hat{y}}_{t+1}^{\left(k^{*}\right)}.$$

The candidate with the lowest validation RMSE is fixed before test evaluation, enabling the adaptive residual-fusion strategy to select the residual-use mode and compensation strength that best match each experimental scenario. Because F0 is included in the candidate set, the framework retains the XGBoost primary prediction whenever none of the residual-use strategies improves validation performance.

The model implementation and evaluation workflow was carried out primarily in Python 3.10.4. The CNN-BiLSTM residual-learning branch was implemented using PyTorch 2.9.0, whereas the XGBoost primary predictor was implemented using XGBoost 1.7.6.

The main model and fusion parameters are summarized in Table 2. XGBoost and CNN-BiLSTM settings were kept consistent across comparable experiments, whereas the candidate-specific fusion coefficients were determined using the validation set. Validation RMSE was used as the primary selection criterion, and the test set was reserved exclusively for final performance evaluation.

#### 2.4.6. Grouped TreeSHAP Attribution and Residual-Correction Gain Analysis

The analysis addresses the two functional stages of the framework. Grouped TreeSHAP quantifies the contributions of physically meaningful feature groups to the XGBoost primary prediction. Residual-correction performance is evaluated using the sample-wise correction gain, MAE reduction, and positive-benefit ratio, which characterize the effectiveness of residual compensation across experimental scenarios.

In the primary prediction branch analysis, the explanation object is the trained XGBoost prediction function. Let the input feature vector of the *i*th test sample be

(35) $$x_{i}=\left[x_{i1},x_{i2},\ldots ,x_{iM}\right].$$

For the trained XGBoost prediction function f(·), SHAP decomposes the model output into a baseline output and individual feature contributions:

(36) $$f\left(x_{i}\right)=\phi_{0}+\sum_{j=1}^{M}\phi_{ij}.$$

Here, $\phi_{0}$ is the expected output under the background sample distribution and $\phi_{ij}$ is the SHAP contribution of feature *j* to the prediction of sample *i*. When $\phi_{ij}>0$, the feature increases the prediction relative to the baseline; when $\phi_{ij}<0$, it decreases the prediction relative to the baseline.

According to the Shapley value definition, the contribution of feature *j* to sample *i* is the weighted marginal contribution across all possible feature subsets:

(37) $$\phi_{ij}=\sum_{S\subseteq F\setminus \{j\}}\frac{|S|!(M-|S|-1)!}{M!}\left[f_{S\cup \{j\}}\left(x_{i}\right)-f_{S}\left(x_{i}\right)\right].$$

Here, F={1,2,…,M} is the complete feature set, *S* is any feature subset not containing feature *j*, and $f_{S}\left(x_{i}\right)$ denotes the conditional expected model output for sample $x_{i}$ given only feature subset *S*. This definition reflects the average marginal influence of feature *j* across different feature combinations. Because XGBoost is a tree ensemble model, TreeSHAP is used to compute feature contributions efficiently and consistently.

TEC prediction inputs contain multiple TEC lag terms, local-time features, the F10.7 solar radio flux, geomagnetic indices, and IMF components. If only individual feature importance is analyzed, the explanation can become overly dispersed. To summarize the model’s information sources according to their physical roles, the input features are divided into five groups: TEC state memory, diurnal cycle, solar activity, geomagnetic activity and interplanetary magnetic field. TEC state memory represents historical persistence and short-term variation of TEC. The diurnal cycle is represented by local-time harmonic factors. Solar activity is mainly represented by F10.7. Geomagnetic activity is represented by Dst and ap indices. The interplanetary magnetic field is represented by Bx, By and Bz components.

At the global explanation level, this study uses the sum of average absolute SHAP values of all features within each feature group to represent the contribution of that feature group to the primary prediction branch, and further calculates normalized contribution ratios to compare changes in the model’s information-use structure across experimental scenarios. The SHAP contribution of feature group *g* is defined as

(38) $$C_{g}=\sum_{j\in G_{g}}\frac{1}{N}\sum_{i=1}^{N}\left|\phi_{ij}\right|.$$

Here, $G_{g}$ is the feature set corresponding to feature group *g*, and *N* is the number of test samples included in the interpretation analysis. To facilitate comparison among experimental scenarios, the normalized feature-group contribution ratio is defined as

(39) $$R_{g}=\frac{C_{g}}{\sum_{k=1}^{K}C_{k}}\times 100\%,$$

where *K* is the number of feature groups and $R_{g}$ is the relative contribution proportion of group *g* in the current scenario. $R_{g}$ represents the normalized relative contribution of feature group *g* to the XGBoost model output in the current scenario.

To compare changes in the model’s information-use structure across different experimental scenarios, pairwise feature-group contribution differences are calculated. Let *r* be the reference scenario and *c* be the comparison scenario. The contribution change of feature group *g* is

(40) $$\Delta R_{g}^{r\to c}=R_{g}^{\left(c\right)}-R_{g}^{\left(r\right)}.$$

When $\Delta R_{g}^{r\to c}>0$, the relative contribution of that feature group is higher in the comparison scenario than in the reference scenario. When $\Delta R_{g}^{r\to c}<0$, the relative contribution is lower. This metric characterizes changes in the model’s information-use structure when moving from univariate to multivariate input, from low to high solar activity, or from geomagnetically quiet to disturbed conditions.

The functional contribution of the residual branch is assessed through the sample-wise reduction in absolute prediction error. For the *i*th test sample, the absolute errors of the XGBoost branch and the final fusion model are defined as follows:

(41) $$e_{i}^{XGB}=\left|y_{i}-{\hat{y}}_{i}^{XGB}\right|,$$

(42) $$e_{i}^{RC}=\left|y_{i}-{\hat{y}}_{i}^{RC}\right|.$$

Here, $y_{i}$ is the observed TEC, ${\hat{y}}_{i}^{XGB}$ is the prediction of the XGBoost branch, and ${\hat{y}}_{i}^{RC}$ is the final prediction of RC-XGB-CNN-BiLSTM. The residual-correction gain is

(43) $$B_{i}=e_{i}^{XGB}-e_{i}^{RC}.$$

When $B_{i}>0$, residual correction reduces the absolute error of the sample. When $B_{i}=0$, the error is unchanged. When $B_{i}<0$, residual correction increases the sample-wise absolute error.

To measure the effectiveness of the validation-constrained adaptive residual-fusion strategy overall, the MAE reduction and positive-benefit ratio are defined as

(44) $$\bar{B}=\frac{1}{N}\sum_{i=1}^{N}B_{i},$$

(45) $$P_{+}=\frac{1}{N}\sum_{i=1}^{N}I\left(B_{i}>0\right)\times 100\%.$$

Here, $\bar{B}$ is the MAE reduction of the fusion model relative to XGBoost, and $P_{+}$ is the positive-benefit ratio. Larger $\bar{B}$ indicates a greater MAE reduction, while higher $P_{+}$ indicates that residual correction benefits a larger proportion of test samples.

### 2.5. Evaluation Metrics

RMSE and MAE are used as the main absolute-error metrics, while P90AE is introduced as a high-quantile diagnostic metric; $R^{2}$ and MAPE are retained as auxiliary metrics. RMSE penalizes large deviations, whereas MAE represents the mean absolute deviation. P90AE is defined in this study as the 90th percentile of the absolute-error distribution to characterize the high-error tail. $R^{2}$ evaluates the ability of the model to represent the overall TEC variation [34], whereas MAPE provides a relative-error perspective [35]. Let *N* be the number of test samples, $y_{i}$ the true value, and ${\hat{y}}_{i}$ the prediction. The metrics are

(46) $$RMSE=\sqrt{\frac{1}{N}\sum_{i=1}^{N}{\left(y_{i}-{\hat{y}}_{i}\right)}^{2}},$$

(47) $$MAE=\frac{1}{N}\sum_{i=1}^{N}\left|y_{i}-{\hat{y}}_{i}\right|,$$

(48) $$P90AE=Q_{0.90}\left(\right|y_{i}-{\hat{y}}_{i}\left|\right).$$

$Q_{0.90}$ denotes the 90% quantile operator. P90AE means that the absolute prediction error of 90% of samples in the test set does not exceed this threshold. Compared with RMSE and MAE, P90AE places greater emphasis on the upper tail of the error distribution and is used to assess robustness for challenging samples, rapid-change intervals, and disturbance-affected cases. In the proposed residual-correction framework, P90AE also assesses whether residual fusion reduces large errors from the primary predictor.

Because RMSE and MAE are absolute error metrics, their values are affected by the magnitude and fluctuation range of TEC. Therefore, comparisons across experimental scenarios should be interpreted together with the corresponding TEC background level, whereas RMSE, MAE, and P90AE comparisons among models within the same scenario directly reflect performance differences. The auxiliary metrics are

(49) $$R^{2}=1-\frac{\sum_{i=1}^{N}{\left(y_{i}-{\hat{y}}_{i}\right)}^{2}}{\sum_{i=1}^{N}{\left(y_{i}-\bar{y}\right)}^{2}},$$

(50) $$MAPE=\frac{100\%}{N}\sum_{i=1}^{N}\frac{|y_{i}-{\hat{y}}_{i}|}{y_{i}}.$$

$R^{2}$ mainly evaluates the ability of the model to explain the overall TEC variation trend. Because TEC sequences often have large dynamic ranges and pronounced periodic structures, a model that captures the main trend may achieve a high $R^{2}$. Thus, $R^{2}$ is not used as the sole criterion. MAPE provides a relative-error perspective, but it can be affected by denominator effects for low TEC values. Therefore, the main conclusions are based on RMSE, MAE and P90AE.

## 3. Experimental Analysis

### 3.1. Effect of Multivariate Inputs on Low-Latitude TEC Prediction

To assess the effect of multivariate inputs on low-latitude TEC prediction, univariate and multivariate experiments are conducted using hourly TEC data at 20° N, 110° E in 2019. The univariate setting uses only historical TEC, whereas the multivariate setting additionally incorporates local-time factors, the F10.7 solar radio flux, geomagnetic indices, and IMF components. All models use the same historical window, prediction horizon, chronological training/validation/test split, and evaluation metrics to ensure a consistent comparison. After sample construction, the dataset contains 8736 valid samples, comprising 6989 training samples, 873 validation samples, and 874 test samples.

**Figure 2:**
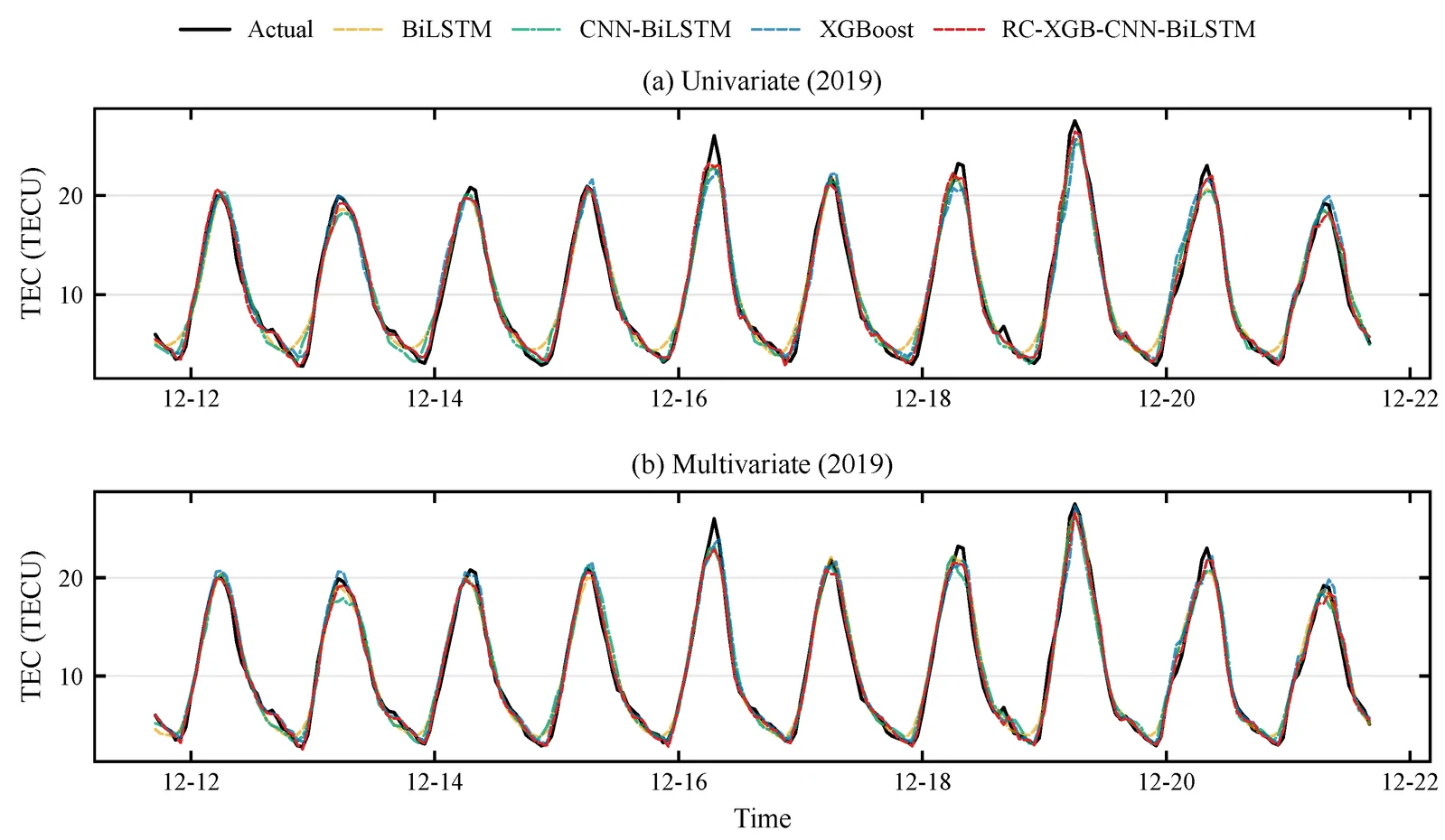
Local enlarged comparison of TEC prediction results under univariate and multivariate input conditions.

Under both input settings, the models reproduce the main diurnal variation of TEC, although noticeable differences remain near peaks, troughs, and rapidly changing intervals (Figure 2). With historical TEC alone, larger amplitude deviations occur around local peaks and intervals with steep temporal gradients. After the multivariate inputs are introduced, the predicted curves more closely follow the observations, particularly near TEC peaks and rapid-change intervals. The improved agreement supports the complementary predictive value of local-time information and solar–terrestrial inputs beyond historical TEC alone.

The quantitative metrics corroborate the curve-level comparison (Table 3). Relative to the univariate setting, all four models achieve lower errors with multivariate inputs. For XGBoost, RMSE decreases from 1.07 to 0.84 TECU, MAE from 0.78 to 0.61 TECU, and P90AE from 1.82 to 1.36 TECU, quantifying the benefit of the added multivariate inputs for the primary predictor.

Under univariate input, XGBoost yields lower errors than BiLSTM and CNN-BiLSTM, whereas RC-XGB-CNN-BiLSTM achieves the lowest values across all four metrics. Its RMSE, MAE, P90AE, and MAPE are 0.77 TECU, 0.59 TECU, 1.26 TECU, and 6.97%, corresponding to reductions of approximately 28.0%, 24.4%, 30.8%, and 17.9% relative to XGBoost. These gains show that residual learning compensates for error components not captured by the primary prediction.

Under multivariate input, RC-XGB-CNN-BiLSTM achieves RMSE, MAE, P90AE, and MAPE values of 0.67 TECU, 0.52 TECU, 1.09 TECU, and 6.20%, respectively. The RMSE is 20.2% lower than that of XGBoost, with concurrent reductions in MAE and P90AE, showing that the improvement extends from the overall error level to the high-error tail.

**Figure 3:**
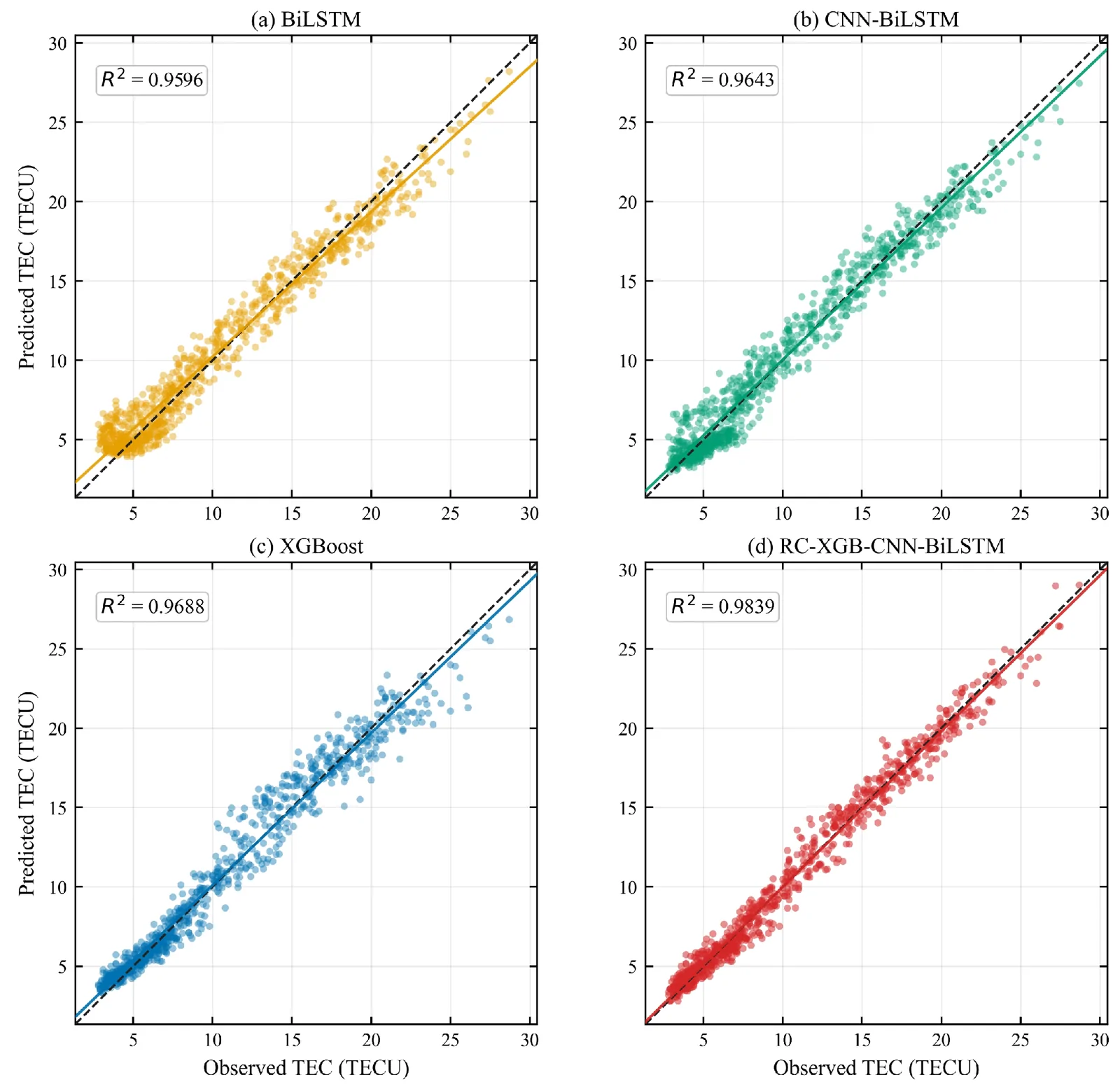
Fitting relationship between predicted and observed TEC for different models under multivariate input conditions.

Under the multivariate setting, the predictions of all four models are concentrated around the 1:1 reference line, indicating that the overall TEC variation is reproduced reasonably well (Figure 3). RC-XGB-CNN-BiLSTM exhibits the most compact scatter distribution, with smaller deviations from the reference line in the medium-to-high TEC range. This reduced dispersion suggests that residual correction mitigates part of the systematic deviation remaining in the XGBoost primary prediction.

**Figure 4:**
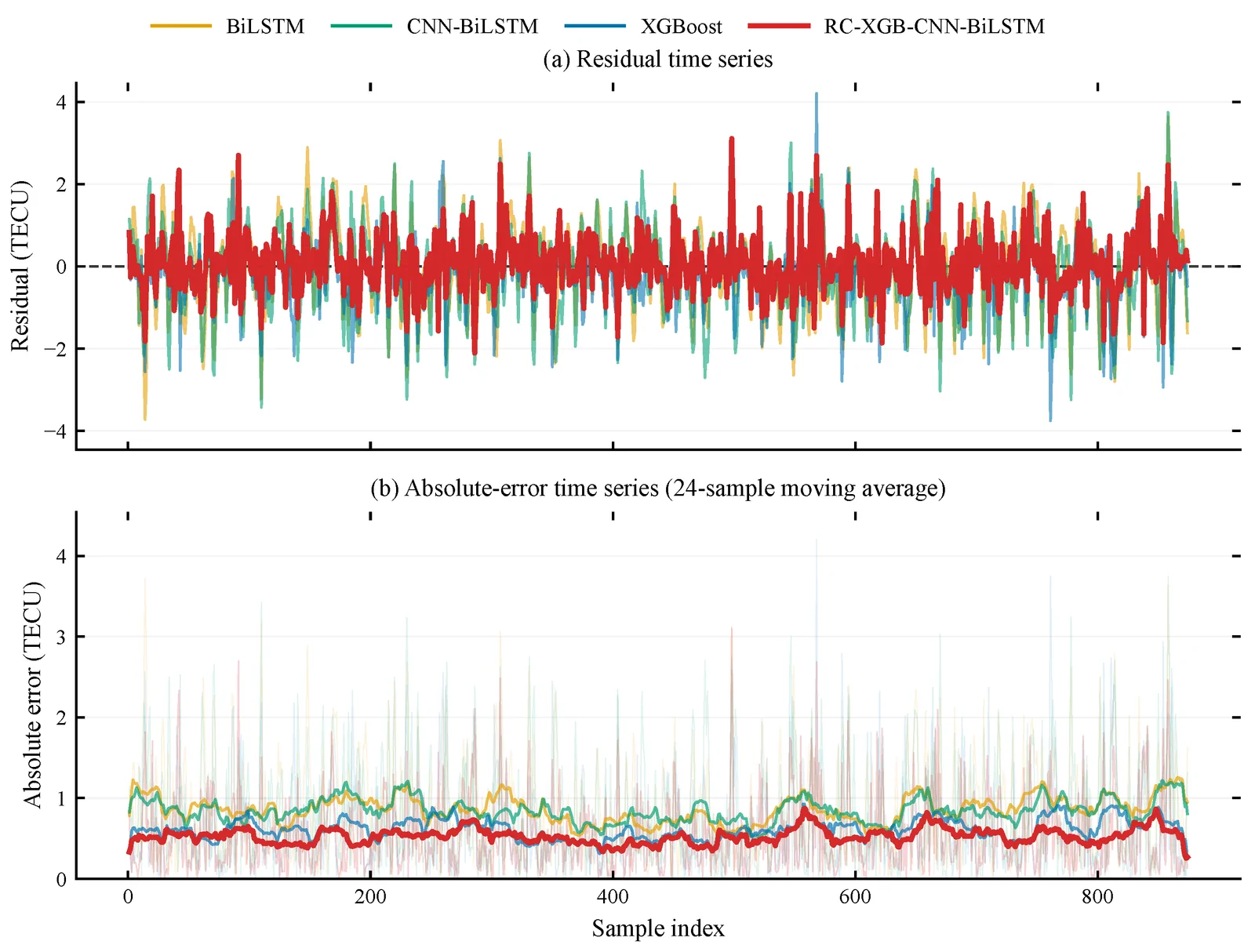
Sample-wise error evolution of the four models in the 2019 multivariate experiment. (**a**) Residual time series. (**b**) Sample-wise absolute errors and their 24-sample moving averages. Light lines denote sample-wise absolute errors, and bold lines denote the corresponding moving averages.

In the 2019 multivariate experiment, RC-XGB-CNN-BiLSTM produces residuals that are more tightly distributed around zero than those of the other models (Figure 4a). Its 24-sample moving-average absolute error also remains lower over most of the test interval (Figure 4b), indicating reduced systematic deviation and greater temporal stability of the sample-wise errors.

**Figure 5:**
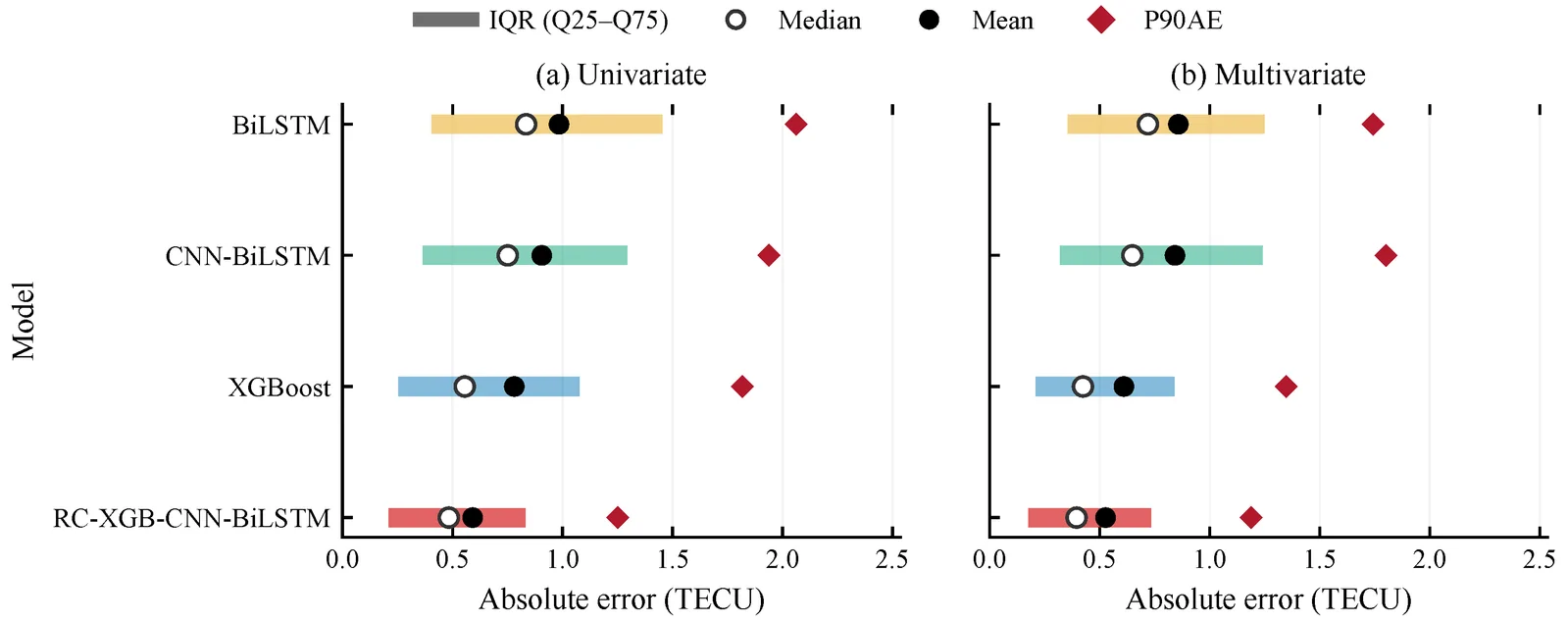
Absolute-error distributions of the four models under (**a**) univariate and (**b**) multivariate input conditions in 2019. Horizontal bars denote the interquartile range (Q25–Q75), while open circles, filled circles and diamonds denote the median, mean and P90AE, respectively.

Distributional statistics provide a complementary assessment of error concentration (Figure 5). Mean, median, interquartile range, and P90AE generally decrease after the additional inputs are introduced. RC-XGB-CNN-BiLSTM exhibits the most compact distribution under both input settings, with lower typical errors and a reduced high-error tail. Together with the sample-wise results in Figure 4, these distributions show that solar–terrestrial inputs improve error stability and that validation-selected residual correction further reduces the remaining errors.

### 3.2. TEC Prediction Under Low and High Solar Activity

**Figure 6:**
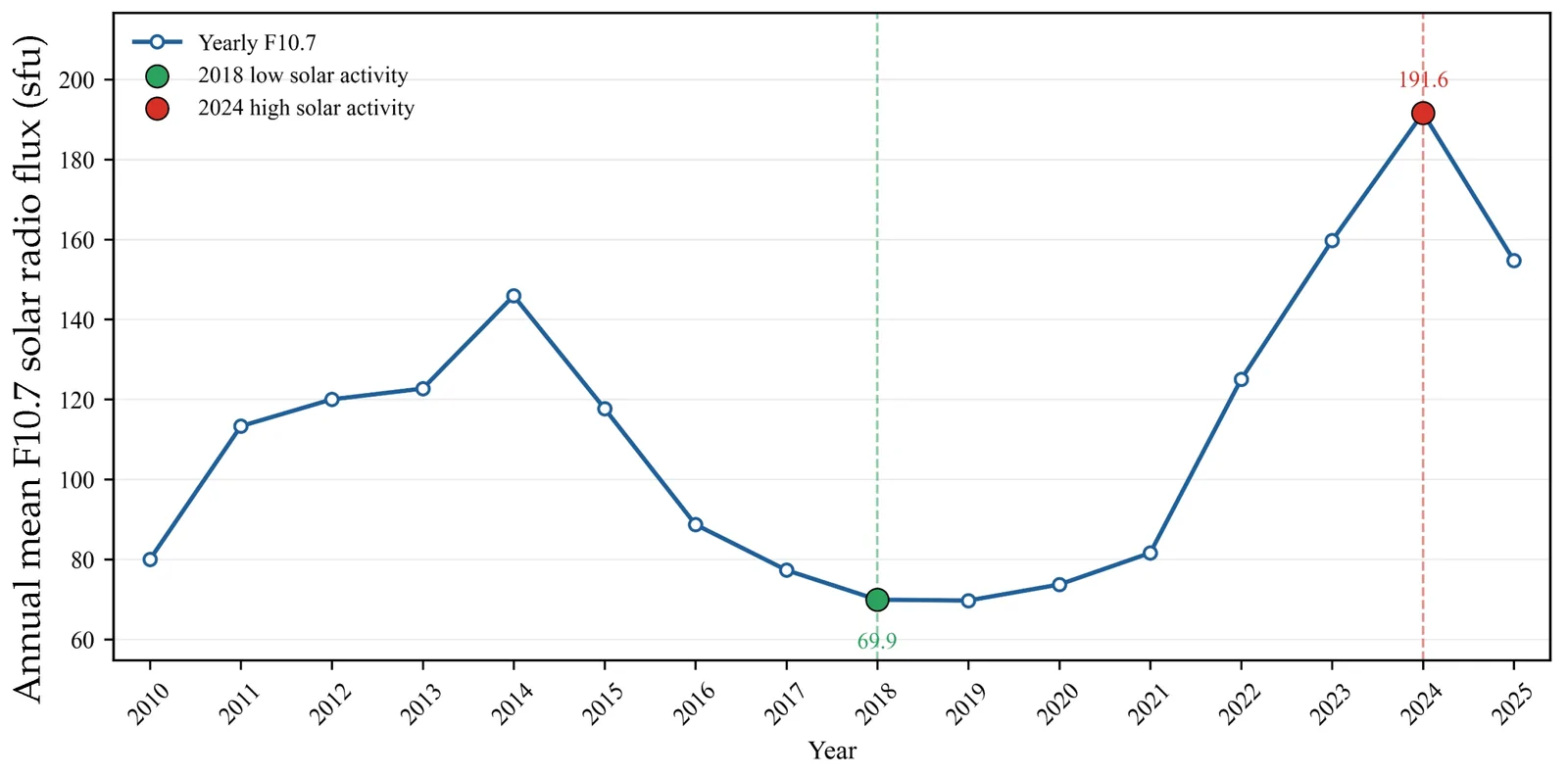
Annual mean F10.7 solar radio flux from 2010 to 2025.

To evaluate model performance under contrasting solar activity conditions, 2018 and 2024 are selected as representative low- and high-activity years, respectively. The annual mean F10.7 solar radio flux confirms the pronounced activity contrast between the two years within 2010–2025 (Figure 6). Because enhanced solar activity generally increases TEC magnitude and short-term variability, the 2024 case represents a more challenging prediction background. After sample construction, the 2018 and 2024 datasets contain 6989/873/874 and 7008/876/876 training/validation/test samples, respectively.

**Figure 7:**
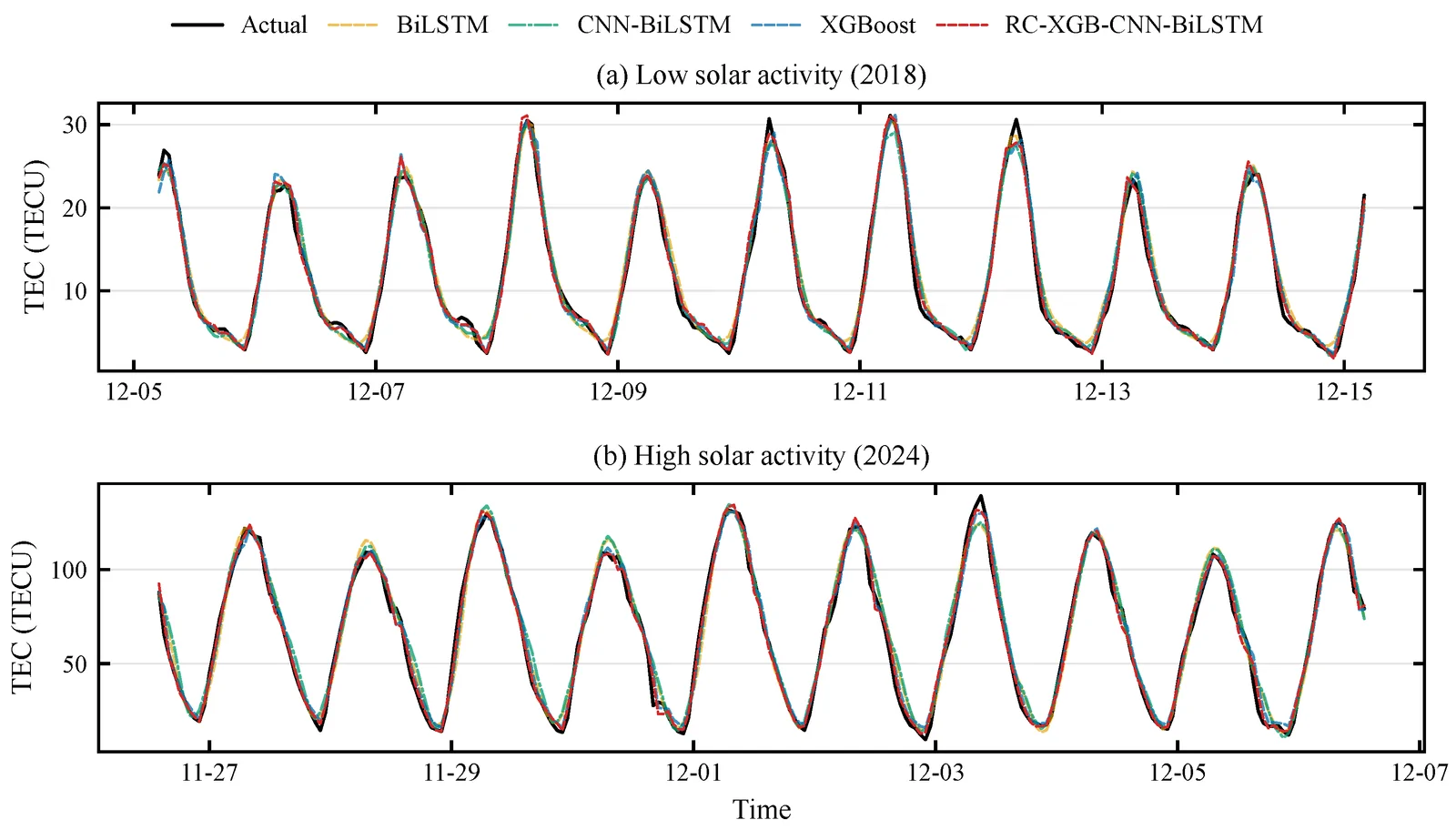
Local enlarged comparison of TEC prediction results under low- and high-solar-activity conditions.

Under low solar activity, TEC exhibits a lower amplitude and a relatively smooth diurnal pattern, and all models reproduce the periodic variation reasonably well (Figure 7a). Under high solar activity, the increased peak magnitude, peak-to-trough variation, and short-term fluctuations reflect stronger TEC nonstationarity (Figure 7b). RC-XGB-CNN-BiLSTM remains closer to the observations than the other models, particularly around local peaks and rapid-change intervals.

**Figure 8:**
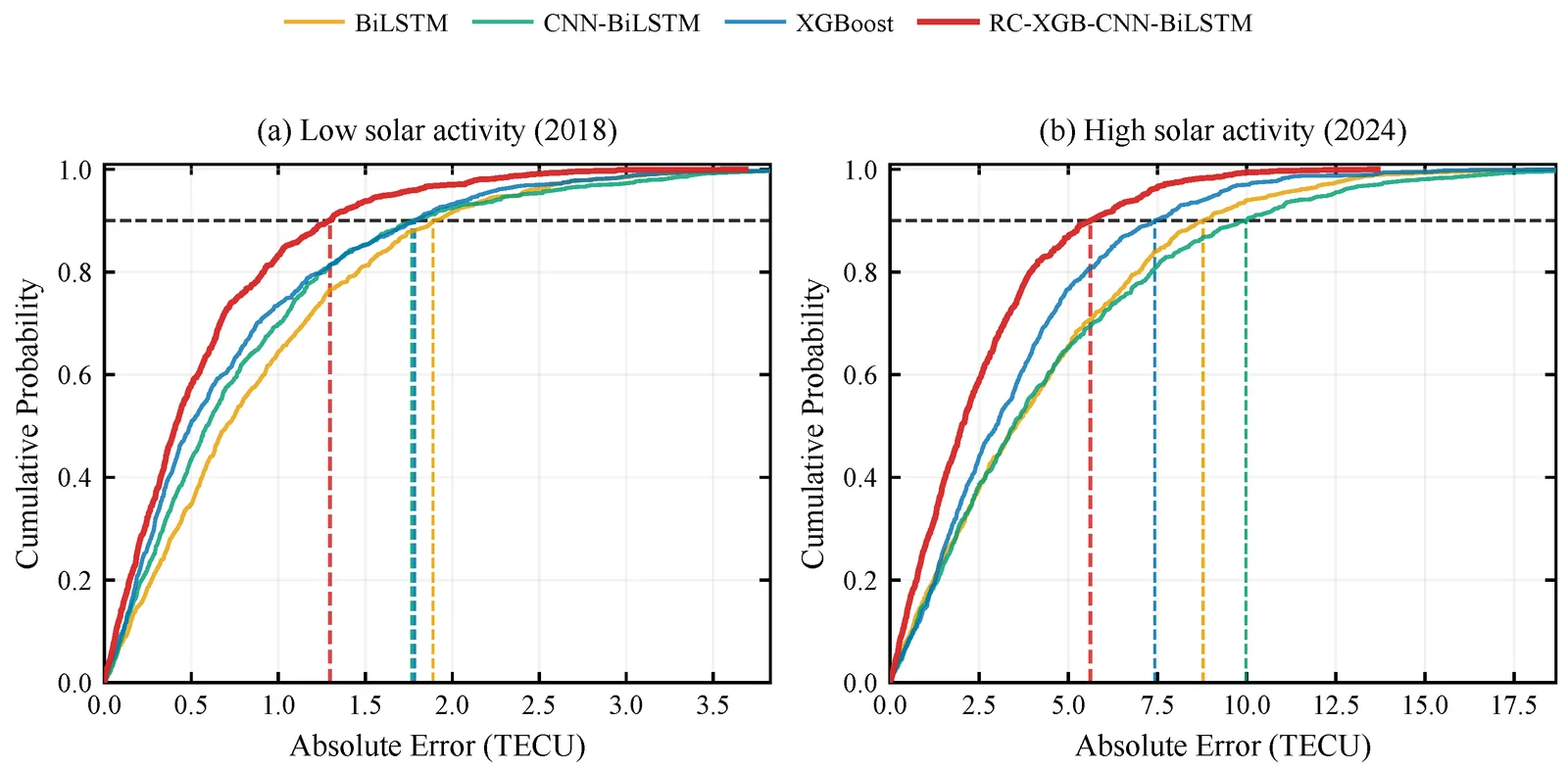
Cumulative distribution functions of absolute errors under low and high solar activity.

The cumulative absolute-error distributions provide a complementary view of model performance across the two solar activity levels (Figure 8). Under low solar activity, the CDFs rise more rapidly and the errors are concentrated within a narrower range, whereas the distributions shift toward larger errors under high solar activity. RC-XGB-CNN-BiLSTM remains closest to the upper-left corner in both cases and yields the lowest P90AE threshold, indicating more effective control of the high-error tail.

The quantitative metrics in Table 4 are consistent with the distributional comparison in Figure 8. In the 2018 low-solar-activity experiment, RC-XGB-CNN-BiLSTM achieves an RMSE of 0.78 TECU, compared with 1.03 TECU for XGBoost, 1.11 TECU for CNN-BiLSTM, and 1.16 TECU for BiLSTM. Relative to XGBoost, RMSE, MAE, and P90AE are reduced by approximately 24.27%, 24.00%, and 27.37%, respectively, showing lower overall and upper-tail errors under the relatively stable low-activity background.

In the 2024 high-solar-activity experiment, absolute errors increase substantially for all models. BiLSTM and CNN-BiLSTM yield RMSE values of 5.39 and 5.90 TECU, respectively, while XGBoost reduces the RMSE to 4.54 TECU and RC-XGB-CNN-BiLSTM further reduces it to 3.36 TECU. Relative to XGBoost, the proposed framework reduces RMSE, MAE, and P90AE by approximately 25.99%, 28.01%, and 24.23%, respectively. Together, the 2018 and 2024 results show that the framework improves prediction under both evaluated solar activity levels, despite the substantially larger absolute errors in 2024.

### 3.3. TEC Prediction Under Quiet and Disturbed Geomagnetic Conditions

**Figure 9:**
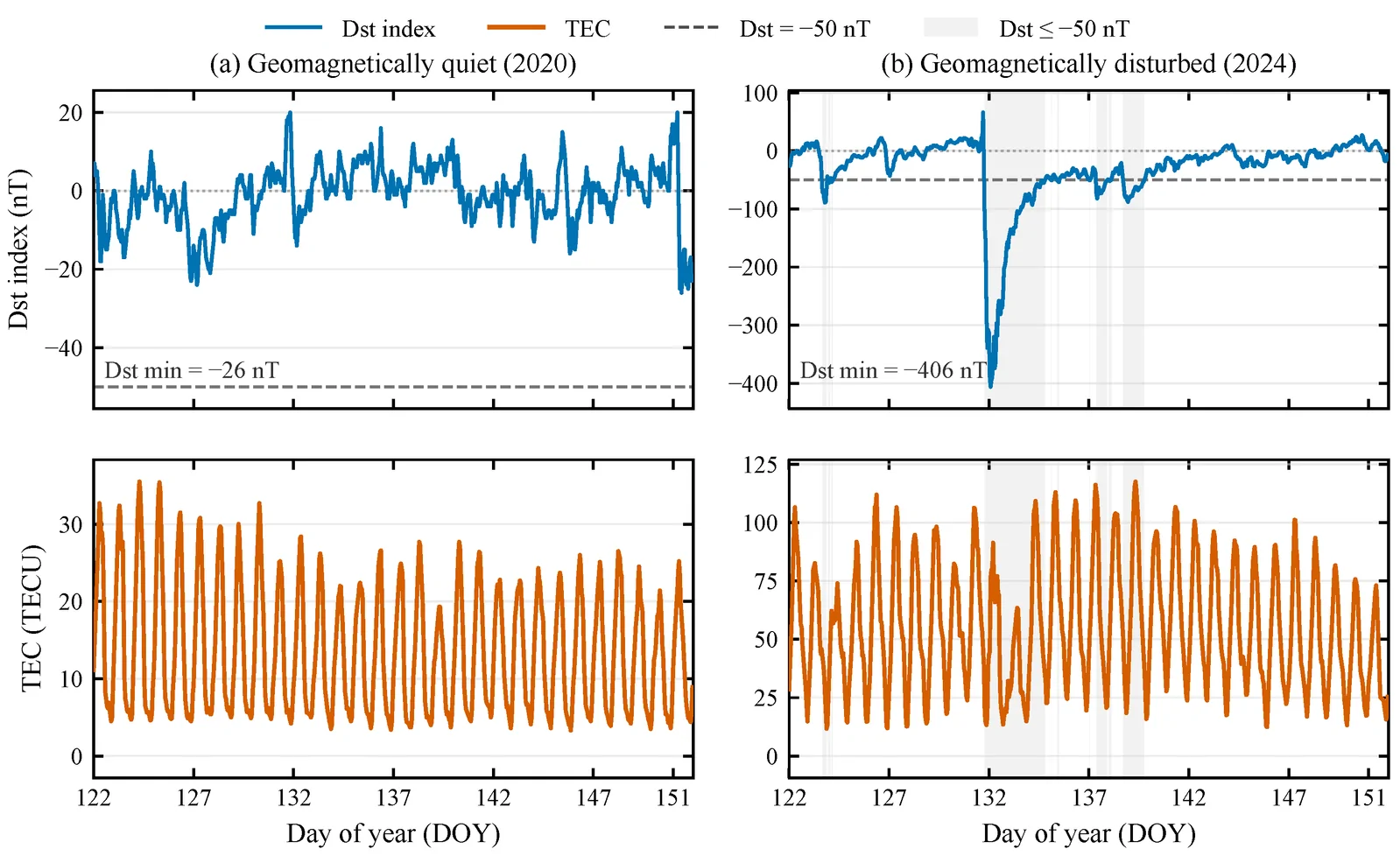
TEC and Dst index variation during DOY 122–151 in 2020 and 2024.

To examine model robustness under contrasting geomagnetic conditions, 30-day periods from DOY 122 to 151 in 2020 and 2024 are selected. Because geomagnetic disturbances are episodic, using full-year samples could dilute disturbance signatures, whereas comparable seasonal windows reduce differences in the seasonal background. During the selected 2020 period, Dst varies weakly and TEC exhibits a lower amplitude and more regular diurnal variation; in contrast, the 2024 period contains pronounced geomagnetic disturbances accompanied by higher TEC levels, larger peaks, and stronger short-term fluctuations (Figure 9). These characteristics provide representative quiet and disturbed backgrounds for evaluating prediction stability. After application of the 24 h historical window, each dataset contains 696 valid samples, including 557 training, 69 validation, and 70 test samples.

Under quiet geomagnetic conditions (Table 5), XGBoost achieves RMSE, MAE, P90AE, and MAPE values of 1.08 TECU, 0.85 TECU, 1.90 TECU, and 7.12%, respectively, all lower than those of BiLSTM and CNN-BiLSTM. The validation procedure selects F0, so the final RC-XGB-CNN-BiLSTM output coincides with the XGBoost primary prediction. The absence of additional residual compensation indicates that the primary branch already provides the best validation performance for the relatively regular TEC variation in the selected quiet period.

Under disturbed geomagnetic conditions (Table 5), prediction errors increase markedly for all models, consistent with the stronger TEC nonstationarity in this period. BiLSTM and CNN-BiLSTM yield RMSE values of 9.66 and 6.99 TECU, respectively, whereas XGBoost reduces the RMSE to 3.53 TECU. RC-XGB-CNN-BiLSTM further reduces RMSE, MAE, P90AE, and MAPE to 3.44 TECU, 2.83 TECU, 5.80 TECU, and 8.69%, respectively. Although the improvement over XGBoost is limited, the lower P90AE indicates that residual correction still reduces part of the high-error tail during disturbed conditions.

### 3.4. Ablation Analysis of Residual Learning and Fusion Strategies

All variants are evaluated using the same 2019 multivariate samples, chronological data partitions, XGBoost primary predictions and out-of-fold residual targets. The compared variants are XGBoost alone (M0), XGBoost with a CNN residual learner (M1), XGBoost with a BiLSTM residual learner (M2), direct CNN-BiLSTM residual addition (M3) and validation-selected CNN-BiLSTM residual fusion (M4).

**Figure 10:**
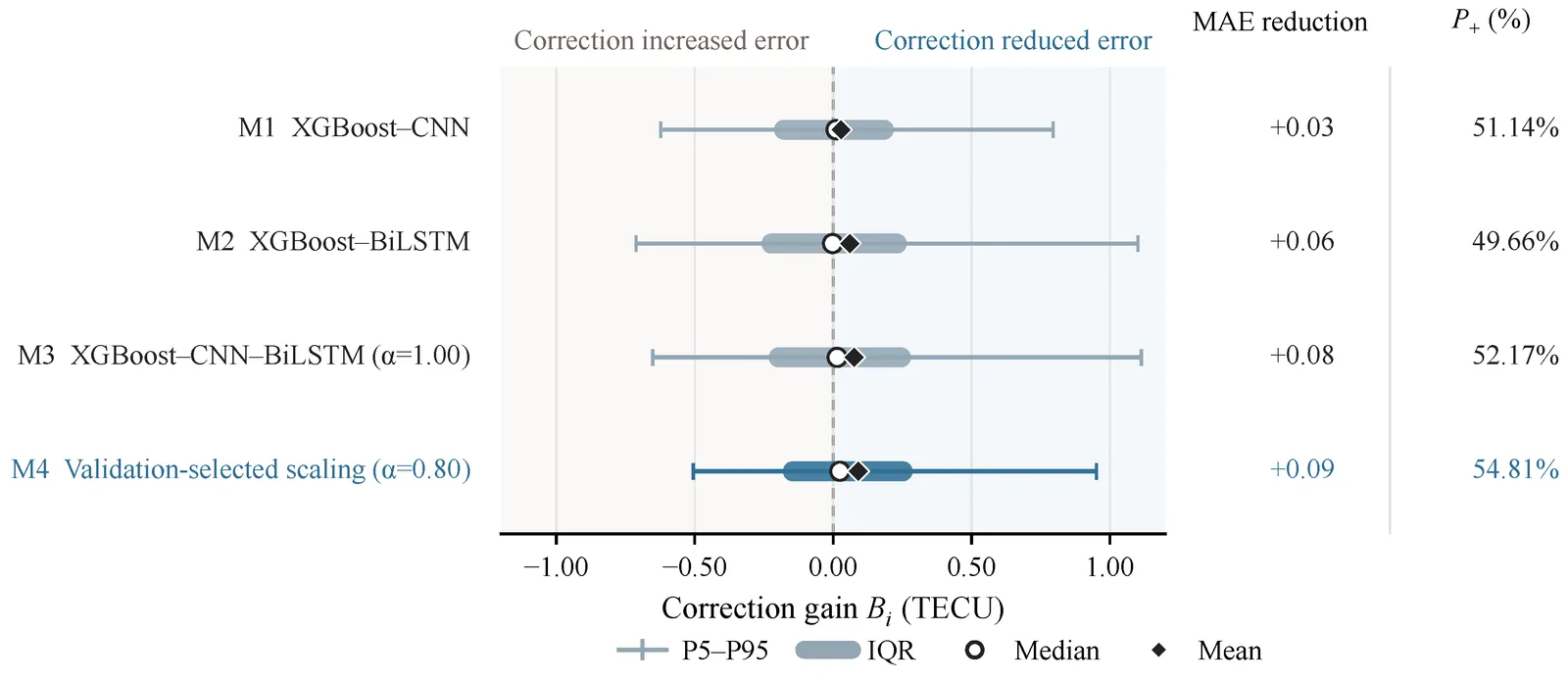
Sample-wise correction gains of the residual-learning variants in the 2019 multivariate experiment. Positive values indicate a reduction in absolute error relative to XGBoost.

The test-set ablation results show a progressive reduction in RMSE from 0.84 TECU for XGBoost alone (M0) to 0.77, 0.71, and 0.69 TECU for M1, M2, and M3, respectively (Table 6). These improvements support the complementary contributions of local fluctuation extraction and bidirectional temporal modeling. M4, which combines CNN-BiLSTM residual learning with validation-selected global scaling ($\alpha_{g}=0.80$), further reduces RMSE to 0.67 TECU and achieves the lowest MAE and P90AE. At the sample level, M4 yields an MAE reduction of 0.09 TECU and a positive-benefit ratio of 54.81%, with the corresponding correction-gain distribution shown in Figure 10.

To determine the final residual-use strategy for the 2019 multivariate experiment, the five candidate outputs are compared on the validation set. F2 with $\alpha_{g}=0.80$ achieves the lowest validation RMSE of 0.72 TECU (Table 7) and is therefore fixed before test evaluation.

### 3.5. Spatial Validation at Multiple Low-Latitude Grid Points

Additional experiments are conducted using 2022 CODE GIM data at 15° N, 110° E; 20° N, 110° E; and 20° N, 75° E. The first two locations provide a latitude contrast at the same longitude, whereas the latter two provide a longitude contrast at the same latitude. Each grid point is modeled independently using its own TEC sequence and the local-time encoding defined in Section 2.1. Each dataset contains 8736 valid samples, divided chronologically into 6989 training samples, 873 validation samples and 874 test samples.

The proposed framework achieves the lowest RMSE, MAE, and P90AE at all three grid points (Table 8). Its RMSE values are 2.90, 2.82 and 2.31 TECU at 15° N, 110° E; 20° N, 110° E; and 20° N, 75° E, corresponding to reductions of 14.5%, 19.9% and 25.0% relative to XGBoost. The improvements observed under both latitude and longitude contrasts support the transferability of the framework across the evaluated low-latitude locations.

### 3.6. Feature-Group Attribution and Residual-Correction Gain Analysis

This section combines grouped TreeSHAP attribution and residual-correction gain analysis to characterize the information used by the primary predictor, examine how attribution patterns vary across solar and geomagnetic activity conditions, and identify the conditions under which residual compensation is effective. The input variables are organized into five physically meaningful groups: TEC state memory, diurnal cycle, solar activity, geomagnetic activity and interplanetary magnetic field.

**Figure 11:**
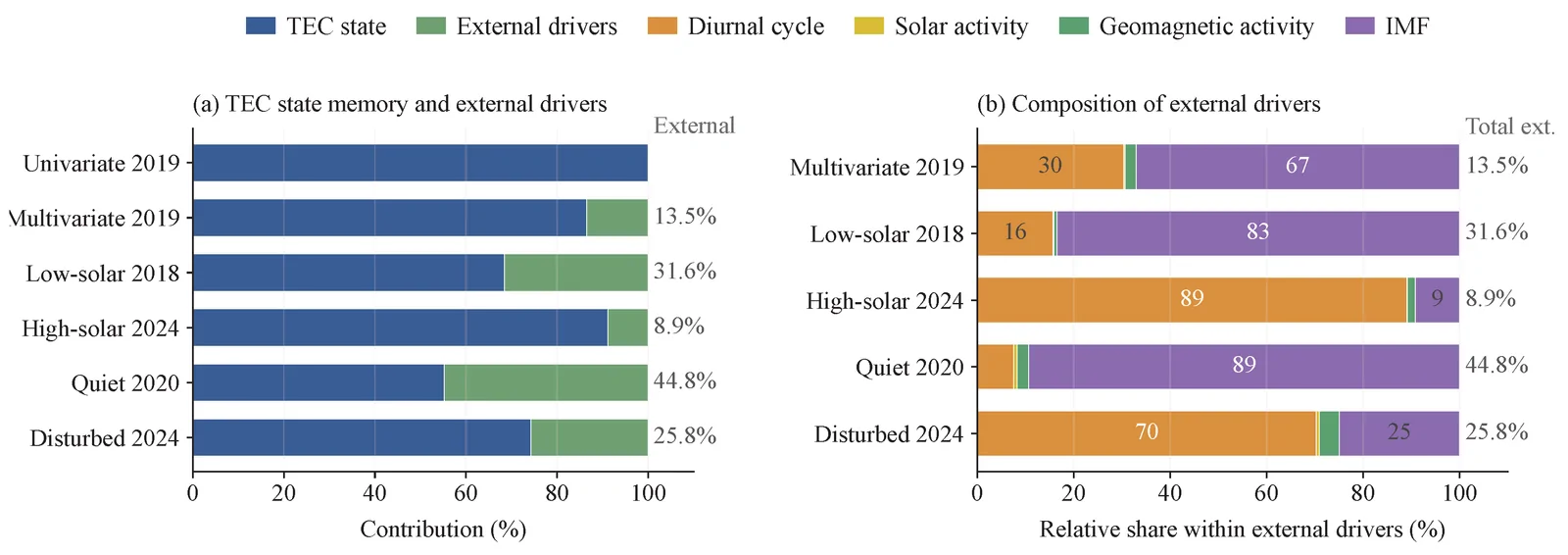
Feature-group SHAP contribution structure under different scenarios.

TEC state memory dominates the grouped attribution across all evaluated scenarios, indicating that short-term TEC prediction relies primarily on the persistence of the recent ionospheric state (Figure 11a). The remaining feature groups provide smaller but activity-dependent contributions, whose internal composition is further resolved in Figure 11b.

In the 2019 univariate experiment (Figure 11a), the attribution is concentrated in TEC state memory because no solar–terrestrial inputs are included, indicating a dominant dependence on persistence and autocorrelation in the historical TEC sequence. With multivariate input, the TEC-state contribution decreases to 86.5%, while the combined contribution of the non-TEC feature groups reaches 13.5%. The corresponding non-TEC contributions are 31.6%, 44.8%, and 25.8% in the 2018 low solar activity, 2020 geomagnetically quiet, and 2024 geomagnetically disturbed cases, respectively. These results indicate that local-time features, the F10.7 solar radio flux, geomagnetic indices, and IMF components provide complementary predictive information whose relative importance varies with the activity background.

Within the non-TEC feature groups, the attribution composition changes substantially across activity conditions (Figure 11b). IMF-related features account for approximately 67% and 83% of the combined non-TEC attribution in the 2019 multivariate and 2018 low-solar-activity cases, respectively. By contrast, diurnal-cycle features account for approximately 89% and 70% in the 2024 high-solar-activity and geomagnetically disturbed cases. These shifts indicate that the relative use of auxiliary predictive information varies with the ionospheric background, with a larger contribution from local-time encoding in the evaluated high-activity cases.

**Figure 12:**
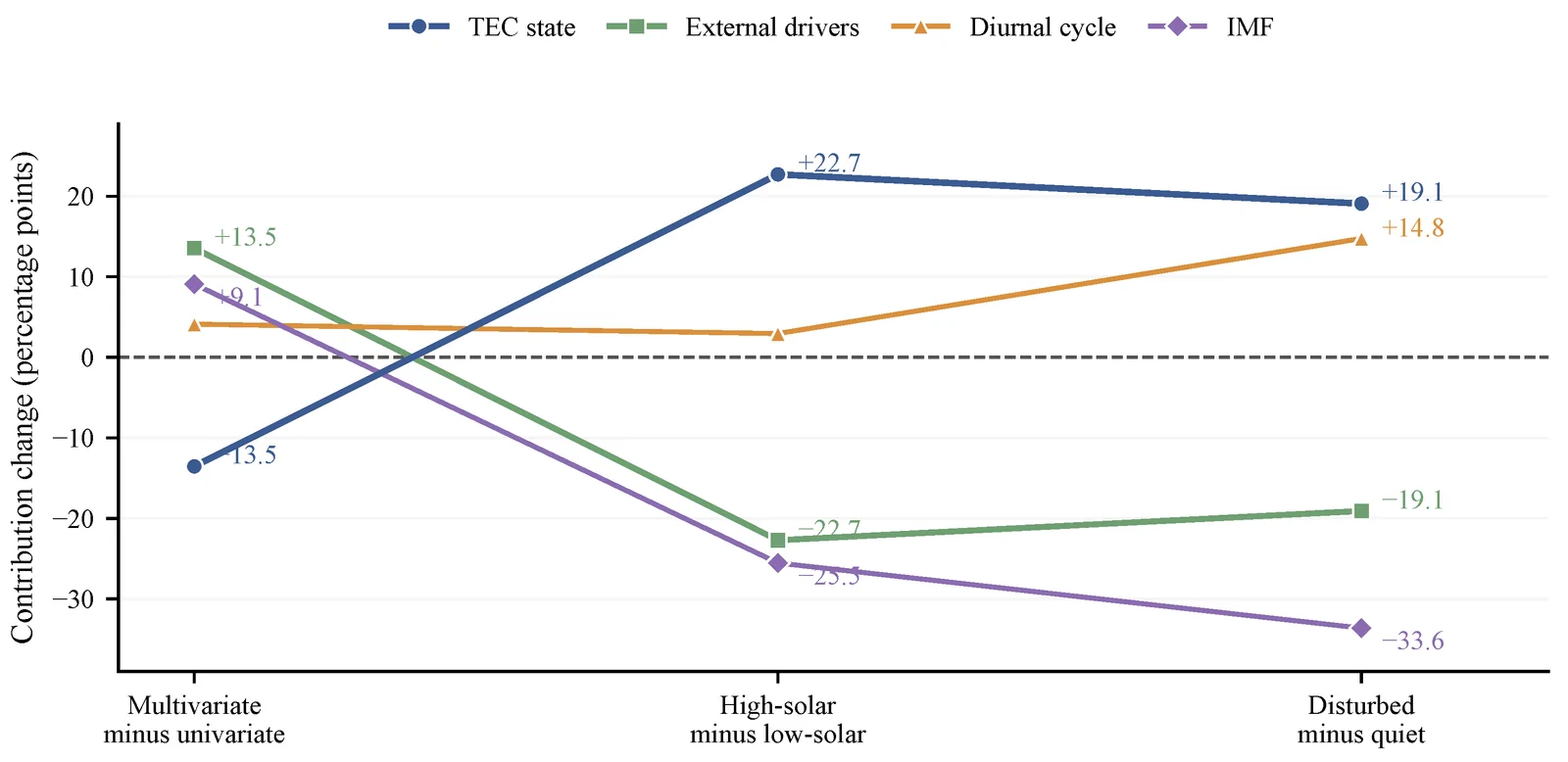
Feature-group SHAP contribution changes under different scenarios.

Scenario-to-scenario changes in grouped attribution are summarized in Figure 12. Relative to the 2019 univariate experiment, the TEC-state contribution decreases by 13.5 percentage points in the multivariate experiment, while the IMF and diurnal-cycle contributions increase by 9.1 and 4.1 percentage points, respectively. From the 2018 low-solar-activity case to the 2024 high-solar-activity case, the TEC-state contribution increases by 22.7 percentage points and the IMF contribution decreases by 25.5 percentage points. Similarly, the disturbed 2024 case shows increases of 19.1 and 14.8 percentage points in the TEC-state and diurnal-cycle contributions relative to the quiet 2020 case, together with a 33.6 percentage-point decrease in IMF contribution. These changes indicate increased reliance on recent TEC state and local-time information within the evaluated high-activity and disturbed backgrounds.

**Figure 13:**
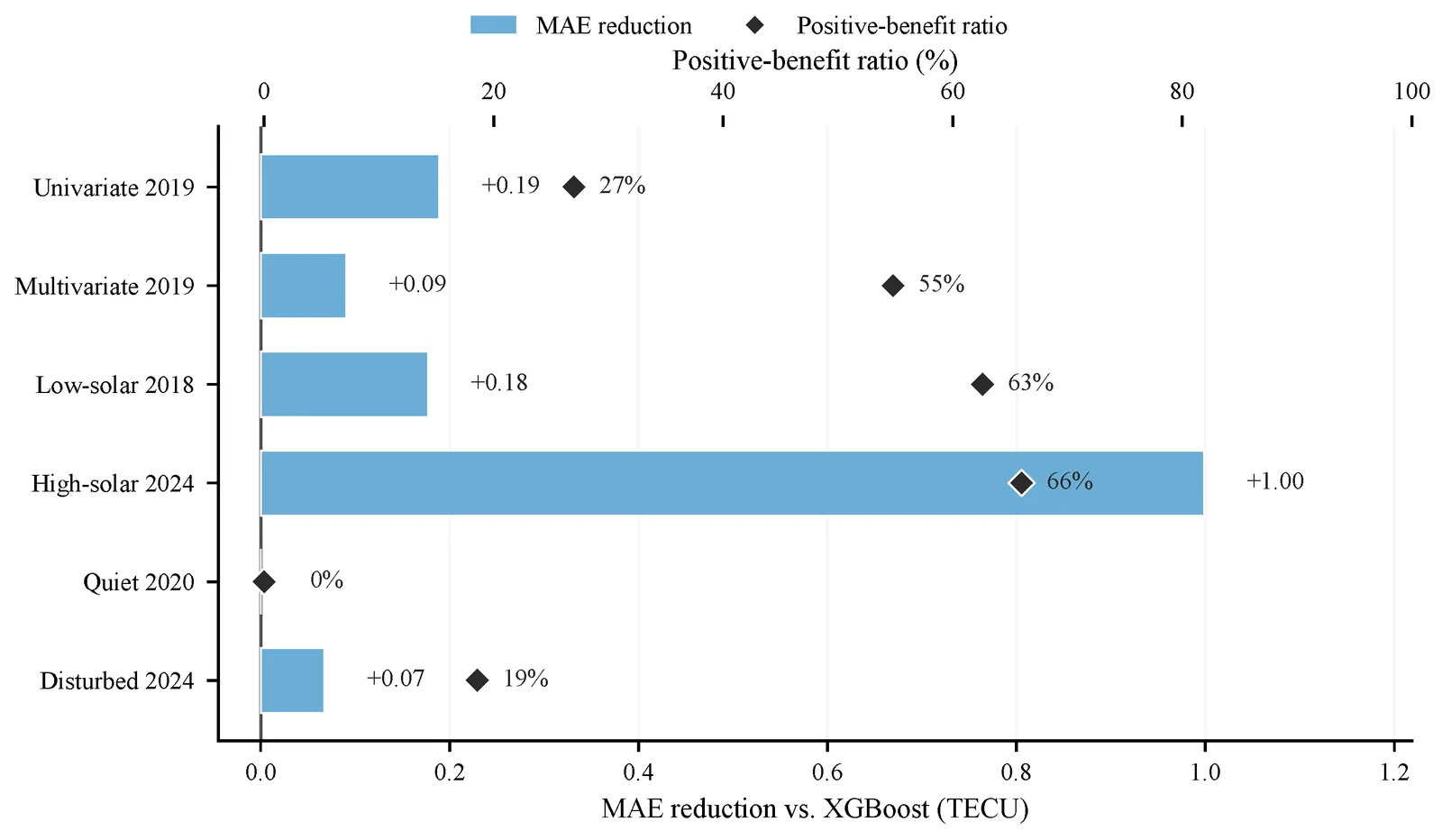
Residual-correction gain analysis across experimental scenarios.

Residual-correction effectiveness varies substantially across the six experimental scenarios (Figure 13). The largest MAE reduction, 1.00 TECU, occurs under high solar activity, with a positive-benefit ratio of 66%. The corresponding MAE reductions are 0.19, 0.09, and 0.18 TECU for the 2019 univariate, 2019 multivariate, and 2018 low solar activity cases, respectively. Under quiet geomagnetic conditions, validation selection retains the XGBoost primary prediction, whereas the disturbed case yields a smaller MAE reduction of approximately 0.07 TECU with a positive-benefit ratio of 19%.

**Figure 14:**
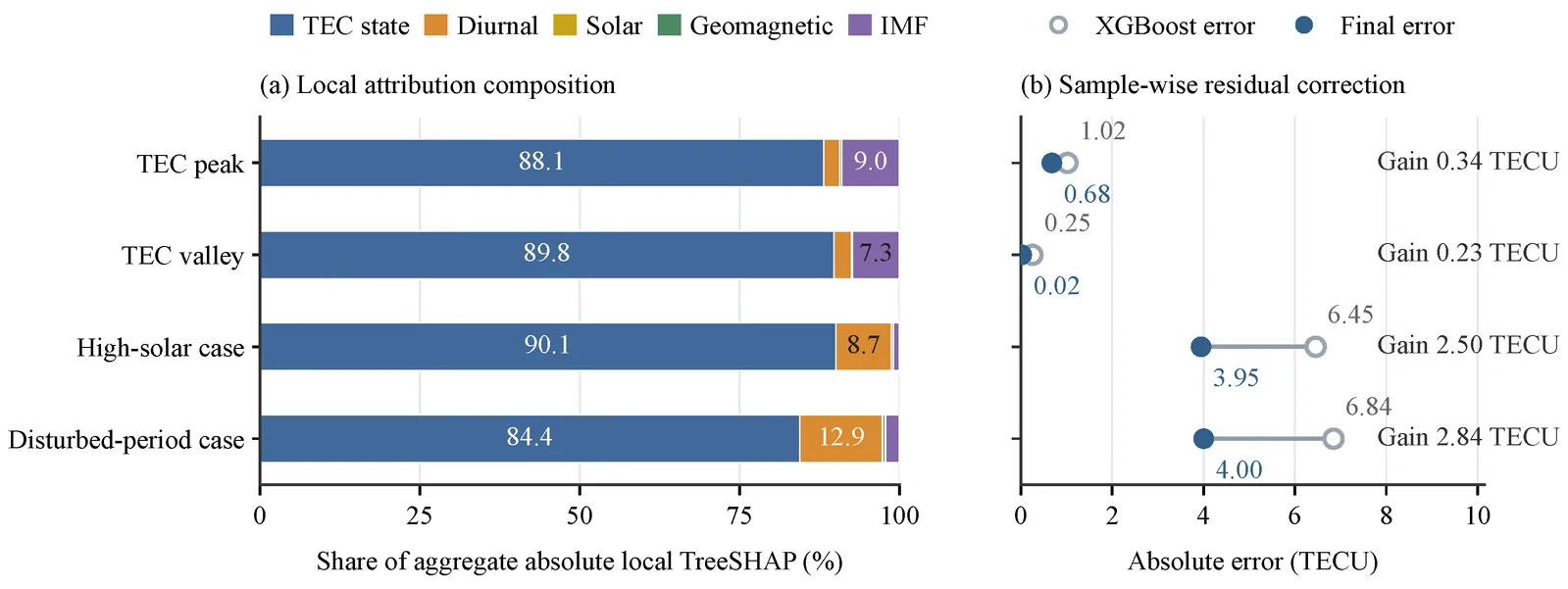
Grouped local attribution and residual-correction results for representative TEC-peak, TEC-valley, high-solar-activity and disturbed-period samples. (**a**) Relative composition of absolute local TreeSHAP values for the XGBoost primary prediction. (**b**) Absolute errors before and after residual correction.

Four representative samples are selected to examine local attribution and residual correction under different TEC and activity conditions. The TEC-peak and TEC-valley cases are drawn from the 2019 multivariate test set, while the high-solar-activity and disturbed-period cases are selected from the corresponding 2024 experiments based on elevated F10.7 and Dst/ap activity, respectively. The four timestamps are 27 December 2019 05:00, 27 November 2019 22:00, 23 December 2024 09:00, and 30 May 2024 12:00. Their local TreeSHAP compositions and absolute errors before and after residual correction are compared in Figure 14.

Taken together, the attribution and correction-gain analyses show that recent TEC state provides the dominant information for the XGBoost primary prediction, while local-time features and solar-terrestrial inputs contribute condition-dependent complementary information. Residual correction provides its largest scenario-level MAE reduction under high solar activity, whereas validation selection retains the primary prediction when no additional correction is beneficial. For the four representative samples in Figure 14, the absolute-error reductions are 0.34, 0.23, 2.50, and 2.84 TECU, respectively. These results distinguish the information used by the primary predictor from the conditions under which the residual branch contributes additional error reduction.

## 4. Discussion

The experiments clarify the distinct roles of the primary and residual stages in the proposed framework. XGBoost captures the dominant nonlinear relationships among recent TEC, local-time harmonic features, and solar–terrestrial inputs, while the out-of-fold residual experiments show that convolution-based local fluctuation extraction and bidirectional temporal modeling capture error structures that are not fully represented by the primary predictor. The additional gain after validation-based fusion selection shows that residual compensation depends on both the residual-use mode and the correction magnitude.

Published results provide useful context for these findings. Liu et al. [11] reported first-hour global TEC RMSEs of 0.86 TECU during quiet periods and 1.27 TECU during storm periods using an LSTM model driven by historical spherical-harmonic coefficients, solar EUV flux, Dst, and hour-of-day information. Zewdie et al. [9] demonstrated low-latitude TEC forecasts up to 5 h ahead after random-forest-based selection of solar–terrestrial parameters, while regional and storm-oriented studies have used ConvLSTM and CNN-BiLSTM architectures to represent spatiotemporal variability [14,16,22]. In the present 2019 multivariate grid-point experiment, RC-XGB-CNN-BiLSTM achieves an RMSE of 0.67 TECU and an MAE of 0.52 TECU, corresponding to a 20.2% RMSE reduction relative to the XGBoost primary predictor under the same samples and chronological split. Because the spatial domain, forecast horizon, TEC product, sampling interval, and activity periods differ among studies, these absolute error values should be interpreted as contextual comparisons rather than a direct cross-study model ranking.

The multi-grid experiments further show that the proposed framework achieves the lowest RMSE, MAE, and P90AE at all three additional low-latitude grid points evaluated in 2022. This result supports robustness across the tested latitude and longitude contrasts, although the present grid-wise evaluation is not equivalent to joint regional-field prediction. The grouped TreeSHAP analysis consistently identifies recent TEC state as the dominant information source, while the relative contributions of local-time features and solar–terrestrial inputs vary with the activity background. Together with the residual-gain analysis, these results indicate that the performance improvement arises from combining a strong structured primary predictor with validation-controlled residual compensation rather than from architectural complexity alone.

The principal limitation appears under geomagnetically disturbed conditions. Storm-oriented TEC studies have explicitly incorporated geomagnetic and solar-wind-related information to represent rapid event-dependent changes [16,17,22,23]. In the present disturbed case, the validation-selected strategy yields an MAE reduction of only approximately 0.07 TECU and a positive-benefit ratio of 19%, whereas under quiet geomagnetic conditions the framework retains the XGBoost primary prediction. This behavior suggests that a fixed 24 h single-grid history does not fully represent storm-time spatial propagation and event-specific forcing, motivating future extensions with disturbance-precursor inputs, neighboring-grid information, event-aware training, and broader seasonal and regional validation.

## 5. Conclusions

This study developed a validation-constrained adaptive residual-fusion framework for one-hour-ahead low-latitude TEC prediction. In the 2019 multivariate experiment, the framework achieved an RMSE of 0.67 TECU, representing a 20.2% reduction relative to XGBoost, while the ablation results showed complementary contributions from CNN-based local fluctuation extraction, BiLSTM temporal modeling, and validation-selected residual scaling. The framework also yielded the lowest RMSE, MAE, and P90AE among the compared models at all three additional low-latitude grid points.

Grouped TreeSHAP identified recent TEC state as the dominant predictive information, with local-time features and solar-terrestrial inputs providing condition-dependent complementary contributions. Residual-gain analysis further showed that the benefit of correction is scenario-dependent: the largest gain occurred under high solar activity, whereas validation selection retained the primary prediction under quiet geomagnetic conditions. The smaller improvement under disturbed geomagnetic conditions highlights the limitation of a fixed 24 h single-grid history. Future work will therefore extend the framework to broader seasonal and regional datasets and incorporate disturbance-precursor and neighboring-grid information for storm-time prediction.

## Figures and Tables

**Table 1:** Input variables used for TEC prediction.

Variable	Description	Original Temporal Resolution
TEC	Historical total electron content observation at the target grid point	1 h
HDs	Sine component of the local-time phase	1 h
HDc	Cosine component of the local-time phase	1 h
Bx	Radial component of the interplanetary magnetic field	1 h
By	Eastward component of the interplanetary magnetic field	1 h
Bz	Vertical component of the interplanetary magnetic field	1 h
Dst	Dst geomagnetic index (ring-current intensity)	1 h
ap	Planetary geomagnetic ap index	3 h
F10.7	10.7 cm solar radio flux	1 d

**Table 2:** Key model and residual-fusion parameters.

Component	Parameter	Setting/Search Range
XGBoost	Number of trees/ maximum depth	80/5
XGBoost	Learning rate/subsample	0.05/0.95
XGBoost	Column subsampling	1.00
CNN-BiLSTM	CNN filters/kernel size	10/2
CNN-BiLSTM	BiLSTM hidden units	16
CNN-BiLSTM	Dropout/learning rate	$0.20/1\times 10^{-3}$
F2 globally scaled residual addition	$\alpha_{g}$	0–1.50, step 0.05
F3 benefit-aware residual weighting	Benefit classifier	XGBoost
F3 benefit-aware residual weighting	$\delta_{0}/\delta_{1}$	0.02 TECU/0.05
F4 condition-triggered residual correction	$\alpha_{c}$	{0, 0.25, 0.50, 0.75, 1.00, 1.50, 2.00}
F4 condition-triggered residual correction	ρ	{0.50,0.65,0.80,0.90}
F4 condition-triggered residual correction	*c*	{0.50,1.00,1.50,2.00,∞}
Candidate selection	Primary criterion	Validation RMSE

**Table 3:** Prediction performance of four models under univariate and multivariate input conditions.

Scenario	Model	RMSE	MAE	P90AE	MAPE (%)
Univariate	BiLSTM	1.22	0.98	2.06	14.21
	CNN-BiLSTM	1.15	0.91	1.94	11.81
	XGBoost	1.07	0.78	1.82	8.49
	RC-XGB-CNN-BiLSTM	0.77	0.59	1.26	6.97
Multivariate	BiLSTM	1.07	0.86	1.74	11.16
	CNN-BiLSTM	1.07	0.84	1.80	10.18
	XGBoost	0.84	0.61	1.36	6.58
	RC-XGB-CNN-BiLSTM	0.67	0.52	1.09	6.20

**Table 4:** Prediction performance of four models under low and high solar activity.

Solar Activity Level	Model	RMSE	MAE	P90AE	MAPE (%)
Low solar activity (2018)	BiLSTM	1.16	0.90	1.89	12.02
	CNN-BiLSTM	1.11	0.81	1.77	9.53
	XGBoost	1.03	0.75	1.79	8.07
	RC-XGB-CNN-BiLSTM	0.78	0.57	1.30	6.35
High solar activity (2024)	BiLSTM	5.39	4.23	8.79	11.44
	CNN-BiLSTM	5.90	4.51	9.99	12.32
	XGBoost	4.54	3.57	7.43	9.92
	RC-XGB-CNN-BiLSTM	3.36	2.57	5.63	6.40

**Table 5:** Prediction performance of four models under quiet and disturbed geomagnetic conditions.

Geomagnetic Condition	Model	RMSE	MAE	P90AE	MAPE (%)
Quiet period	BiLSTM	2.76	2.06	5.05	17.95
	CNN-BiLSTM	1.82	1.34	3.09	11.42
	XGBoost	1.08	0.85	1.90	7.12
	RC-XGB-CNN-BiLSTM	1.08	0.85	1.90	7.12
Disturbed period	BiLSTM	9.66	8.34	15.80	23.45
	CNN-BiLSTM	6.99	5.91	11.83	16.39
	XGBoost	3.53	2.89	6.06	8.90
	RC-XGB-CNN-BiLSTM	3.44	2.83	5.80	8.69

**Table 6:** Ablation results of residual-learning and fusion components in the 2019 multivariate experiment.

Variant	Description	RMSE	MAE	P90AE	MAPE (%)	MAE Reduction	Positive-Benefit Ratio (%)
M0	XGBoost	0.84	0.61	1.36	6.58	–	–
M1	XGBoost + CNN residual	0.77	0.58	1.29	6.66	0.03	51.14
M2	XGBoost + BiLSTM residual	0.71	0.55	1.16	6.38	0.06	49.66
M3	XGBoost + CNN-BiLSTM residual, α=1	0.69	0.53	1.14	6.39	0.08	52.17
M4	CNN-BiLSTM residual learning with validation-selected global scaling ($\alpha_{g}=0.80$)	0.67	0.52	1.09	6.20	0.09	54.81

**Table 7:** Validation-set performance of candidate residual-fusion strategies in the 2019 multivariate experiment.

Candidate	Residual-Use Strategy	Setting	RMSE	MAE	P90AE
F0	Primary XGBoost prediction without residual correction	–	0.98	0.72	1.66
F1	Direct residual addition	α=1.00	0.74	0.56	1.23
F2	Globally scaled residual addition	$\alpha_{g}=0.80$	0.72	0.55	1.15
F3	Benefit-aware residual weighting	Validation-selected	0.73	0.56	1.17
F4	Condition-triggered residual correction	Validation-selected	0.74	0.56	1.25

**Table 8:** Prediction performance at three low-latitude grid points in 2022.

Grid Point	Model	RMSE	MAE	P90AE
15° N, 110° E	BiLSTM	3.59	2.89	5.86
	CNN-BiLSTM	3.44	2.76	5.52
	XGBoost	3.39	2.69	5.64
	RC-XGB-CNN-BiLSTM	2.90	2.28	4.86
20° N, 110° E	BiLSTM	3.72	3.02	6.01
	CNN-BiLSTM	3.66	2.84	5.88
	XGBoost	3.52	2.82	5.96
	RC-XGB-CNN-BiLSTM	2.82	2.21	4.73
20° N, 75° E	BiLSTM	3.31	2.66	5.44
CNN-BiLSTM		3.24	2.53	5.32
XGBoost		3.08	2.45	5.19
RC-XGB-CNN-BiLSTM		2.31	1.77	3.71

## Data Availability

Publicly available datasets were analyzed in this study. The final CODE Global Ionosphere Map (GIM) products in IONEX format were obtained from the IGS ionosphere product archive hosted by NASA CDDIS at https://cddis.nasa.gov/archive/gnss/products/ionex/ (accessed on 20 March 2026). The F10.7 solar radio flux, Dst and ap geomagnetic indices, and IMF components were obtained from the NASA/GSFC Space Physics Data Facility OMNIWeb at https://omniweb.gsfc.nasa.gov/ (accessed on 7 April 2026). The processed datasets and model code are not publicly available at present, as they comprise study-specific derivative materials generated within the analysis workflow and have not been prepared for public release.
